# Advancements in MV_2_O_6_-Based Particulate Systems for Solar-Light Water Splitting

**DOI:** 10.3390/mi17080904

**Published:** 2026-07-29

**Authors:** Parnapalle Ravi, Jin-Seo Noh

**Affiliations:** Department of Physics and Semiconductor Science, Gachon University, 1342 Seongnamdaero, Sujeong-gu, Seongnam-si 13120, Gyeonggi-do, Republic of Korea

**Keywords:** MV_2_O_6_ metavanadates, solar water splitting, photoelectrochemical water splitting, defect engineering, charge separation

## Abstract

The development of efficient visible-light-driven semiconductor photocatalysts is essential for scalable and sustainable green hydrogen production. Among ternary metal oxides, MV_2_O_6_ (M = Zn, Ni, Cu, Mn, Co, etc.) metavanadates have attracted considerable interest because of their narrow band gaps (~1.8–2.5 eV), strong visible-light absorption, and unique edge-sharing VO_6_ octahedral framework that promotes charge separation. This review summarizes recent advances in the design, synthesis, and electronic engineering of MV_2_O_6_-based photocatalysts for solar water splitting. Since direct particulate overall water splitting has only been demonstrated for MnV_2_O_6_, whereas ZnV_2_O_6_, NiV_2_O_6_, and CuV_2_O_6_ have mainly been investigated as photoelectrodes, both particulate photocatalytic and photoelectrochemical (PEC) systems are critically examined. The review clearly distinguishes these two configurations, highlighting how PEC studies provide valuable insights into charge transport, interfacial processes, and reaction kinetics while recognizing the additional challenges associated with suspension-based photocatalysis. Fundamental crystal structures, electronic band alignments, and charge-transfer characteristics of MV_2_O_6_ compounds are discussed, followed by recent advances in synthesis strategies, including hydrothermal, sol–gel, and deep eutectic solvent (DES)-assisted methods, together with morphology and defect engineering. Particular attention is given to oxygen-vacancy formation and its influence on visible-light absorption and charge separation. Modification strategies, including elemental doping, cocatalyst loading, and the construction of Z-scheme and step-scheme (S-scheme) heterojunctions, are critically evaluated for improving photocatalytic efficiency. Finally, the review discusses the key challenges that limit practical applications, including unfavorable band-edge positions, rapid carrier recombination, sluggish surface reaction kinetics, photostability, and the need to establish composition–structure–activity relationships. Future perspectives emphasize rational materials design through advanced characterization, theoretical calculations, and scalable synthesis approaches to accelerate the development of efficient MV_2_O_6_ photocatalysts for solar-driven hydrogen production.

## 1. Introduction

The rapid increase in global energy consumption, coupled with concerns regarding climate change, has accelerated the search for sustainable energy technologies [1,2,3]. Currently, the global energy infrastructure remains heavily dependent on fossil fuels, which contribute significantly to greenhouse gas emissions [4,5,6]. Among renewable energy carriers, hydrogen has emerged as a promising alternative due to its high gravimetric energy density (142 MJ kg^−1^) and clean combustion products [7,8]. However, most industrial hydrogen production still relies on steam methane reforming, resulting in substantial CO_2_ emissions [9]. Consequently, developing green hydrogen generation pathways has become a critical priority.

**Figure 1:**
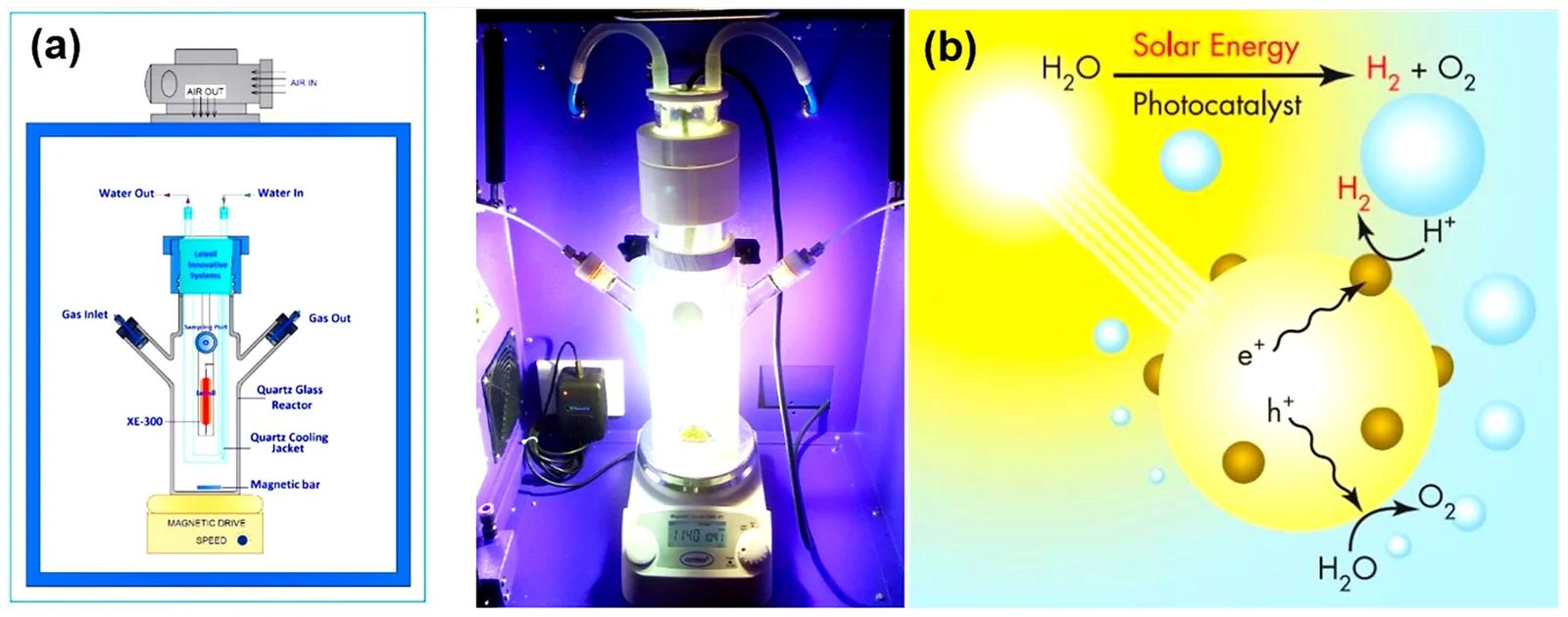
(**a**) Schematic representation of the global energy transition from conventional fossil-fuel-based energy systems toward sustainable solar-driven green hydrogen production, (open access) [19]. (**b**) Illustration of particulate photocatalytic water splitting, highlighting visible-light absorption, photogenerated charge carrier separation and migration, hydrogen evolution reaction (HER), and oxygen evolution reaction (OER) for solar-to-hydrogen conversion (source accessed on 10 June 2026: https://watsonsolar.binghamton.edu/about_solar.html?utm).

Solar-driven photocatalytic water splitting represents an attractive approach for sustainable hydrogen production, directly converting solar energy into chemical fuel [10,11,12,13]. Since Fujishima and Honda’s pioneering work on TiO_2_ electrodes in 1972 [5,14], extensive efforts have focused on developing semiconductor photocatalysts for water splitting. As illustrated in Figure 1a, the transition from fossil-fuel-based systems to solar-driven green hydrogen production via photocatalysis offers a viable pathway toward carbon neutrality. Upon photon absorption, electrons excite from the valence band to the conduction band, generating electron–hole pairs that migrate to the surface, where electrons reduce protons to H_2_ and holes oxidize water to O_2_ [15]. Figure 1b provides a schematic overview of this particulate photocatalytic water splitting process, including light absorption, charge separation, and the hydrogen and oxygen evolution reactions. Among various configurations, particulate photocatalytic systems have attracted considerable attention due to their simple reactor design, low cost, and scalability [11,16,17]. Unlike photoelectrochemical systems requiring external circuitry, particulate photocatalysts operate in slurry reactors under direct solar irradiation [18].

However, the absence of external bias requires that semiconductor band edges straddle the water redox potentials—a stringent requirement few single-phase materials satisfy [20,21].

Despite progress, practical realization of efficient particulate water splitting remains challenging due to insufficient visible-light absorption, rapid electron–hole recombination (picosecond to nanosecond timescale), and sluggish surface kinetics. Traditional wide-bandgap oxides such as TiO_2_ (Eg ≈ 3.2 eV) utilize less than 5% of the solar spectrum. Since visible light (~400–700 nm) accounts for nearly 45% of solar irradiance, developing visible-light-active semiconductors is imperative [22,23].

Over the past decade, several comprehensive review articles have systematically summarized the broader field of particulate photocatalysis and visible-light-active materials. For instance, Jia et al. (2024) provided an extensive overview of metal oxide photoelectrodes, including TiO_2_, ZnO, WO_3_, Fe_2_O_3_, and BiVO_4_, with emphasis on three-dimensionally ordered macroporous (3DOM) structures for enhanced light harvesting and charge transport [24]. Pan and co-workers (2016) published a critical review on defect engineering in oxide photocatalysts, comprehensively summarizing how oxygen vacancies and non-stoichiometric defects modulate electronic band structures, extend visible-light absorption, and create active sites for surface reactions [25]. However, their analysis focused predominantly on binary oxides and well-studied ternary systems such as BiVO_4_, without extending to the less-explored ternary vanadate family. More recently, a review examined the transition from Z-scheme to S-scheme heterojunction architectures for photocatalytic hydrogen evolution, providing fundamental insights into interfacial charge-transfer mechanisms and the preservation of strong redox potentials [26]. Nevertheless, the survey of heterojunction systems was primarily confined to sulfides, nitrides, and carbon-based composites, with only cursory attention to metal vanadates. Regarding vanadate materials specifically, existing review articles have largely concentrated on sodium vanadates for energy storage applications, offering detailed analyses of their crystal structures and electrochemical properties, yet these works do not address photocatalytic water splitting.

Consequently, to our knowledge, no previous review article has specifically focused on the MV_2_O_6_ (M = Zn, Ni, Cu, Mg, Mn, Co) metavanadate family as a distinct class of particulate photocatalysts for solar hydrogen production. The existing literature lacks a systematic comparison of how their crystal structures—particularly the edge-sharing VO_6_ octahedral frameworks—together with their synthetic routes and surface defect chemistries, collectively govern photocatalytic performance. Furthermore, the critical assessment of stability issues, such as photocorrosion in CuV_2_O_6_ and NiV_2_O_6_, and the rational design of metavanadate-based heterojunctions for stoichiometric overall water splitting remain fragmented across individual research papers. Given the rapid proliferation of studies on these materials in recent years, a dedicated, critical review is urgently needed to consolidate these advances, identify structure–activity relationships, and outline a clear roadmap for future development.

**Figure 2:**
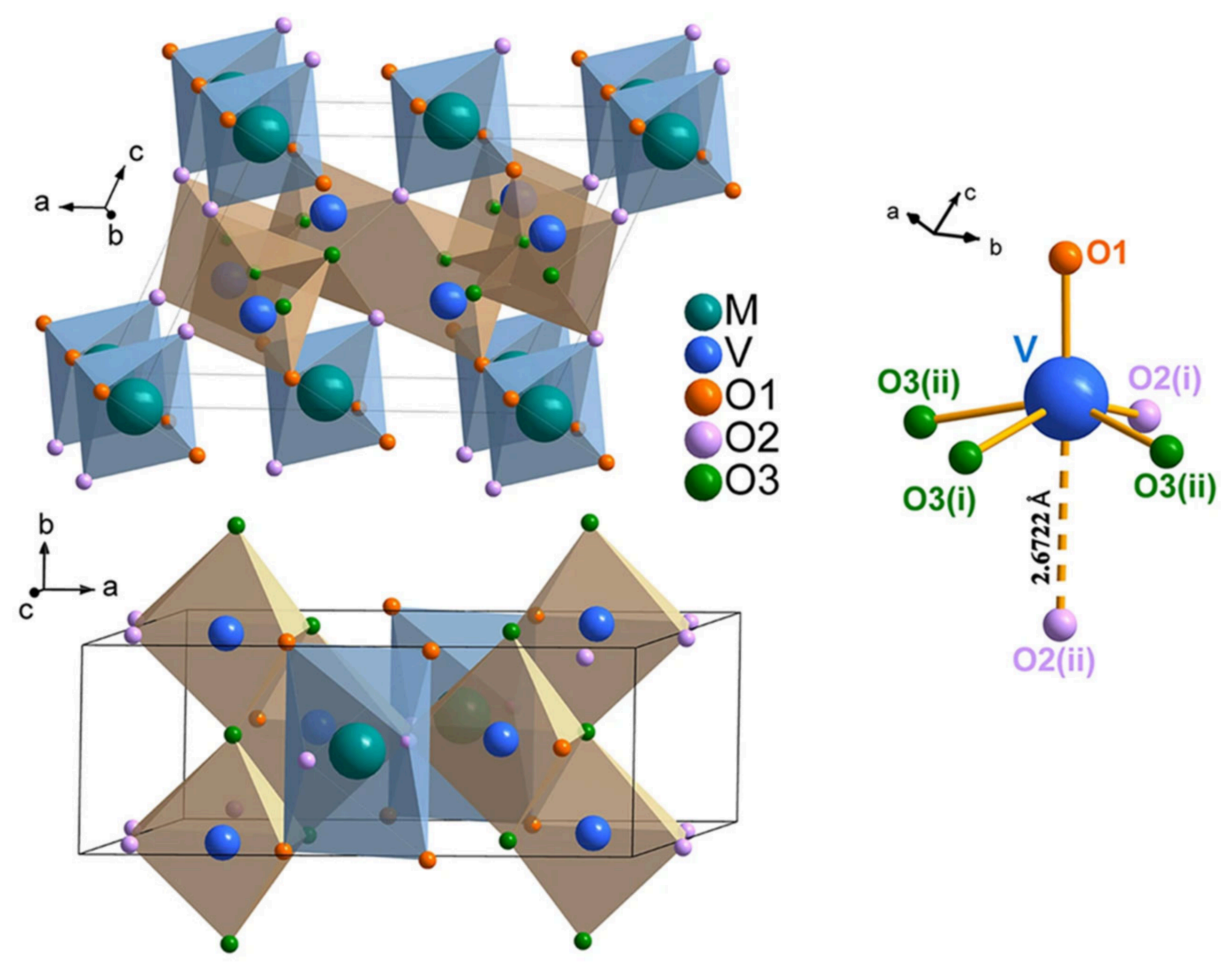
Crystal structures and representative morphologies of MV_2_O_6_ metavanadates (ZnV_2_O_6_, NiV_2_O_6_, CuV_2_O_6_, MgV_2_O_6_, MnV_2_O_6_, and CoV_2_O_6_), (open access) [31].

Among emerging visible-light-responsive photocatalysts, ternary metal vanadates have attracted increasing attention [27,28]. In particular, MV_2_O_6_ (M = Zn, Ni, Cu, Mg, Mn, Co) metavanadates possess narrow band gaps (1.8–2.5 eV), enabling efficient visible-light harvesting [8,29,30,31,32]. Figure 2 presents the crystal structures of representative metavanadates (ZnV_2_O_6_, NiV_2_O_6_, CuV_2_O_6_, MgV_2_O_6_, MnV_2_O_6_, and CoV_2_O_6_) alongside their characteristic morphologies, highlighting the edge-sharing VO_6_ octahedral frameworks that distinguish them from conventional oxides.

Unlike orthovanadates containing isolated VO_4_ tetrahedra, metavanadates with edge-sharing VO_6_ networks often exhibit enhanced electronic delocalization, which can improve charge transport. However, their band-edge positions vary substantially, and while some compositions such as ZnV_2_O_6_ are reported to provide sufficient reducing power for proton reduction, others such as CuV_2_O_6_ require band-structure engineering or heterojunction construction to drive water reduction effectively [8,29,33].

**Figure 3:**
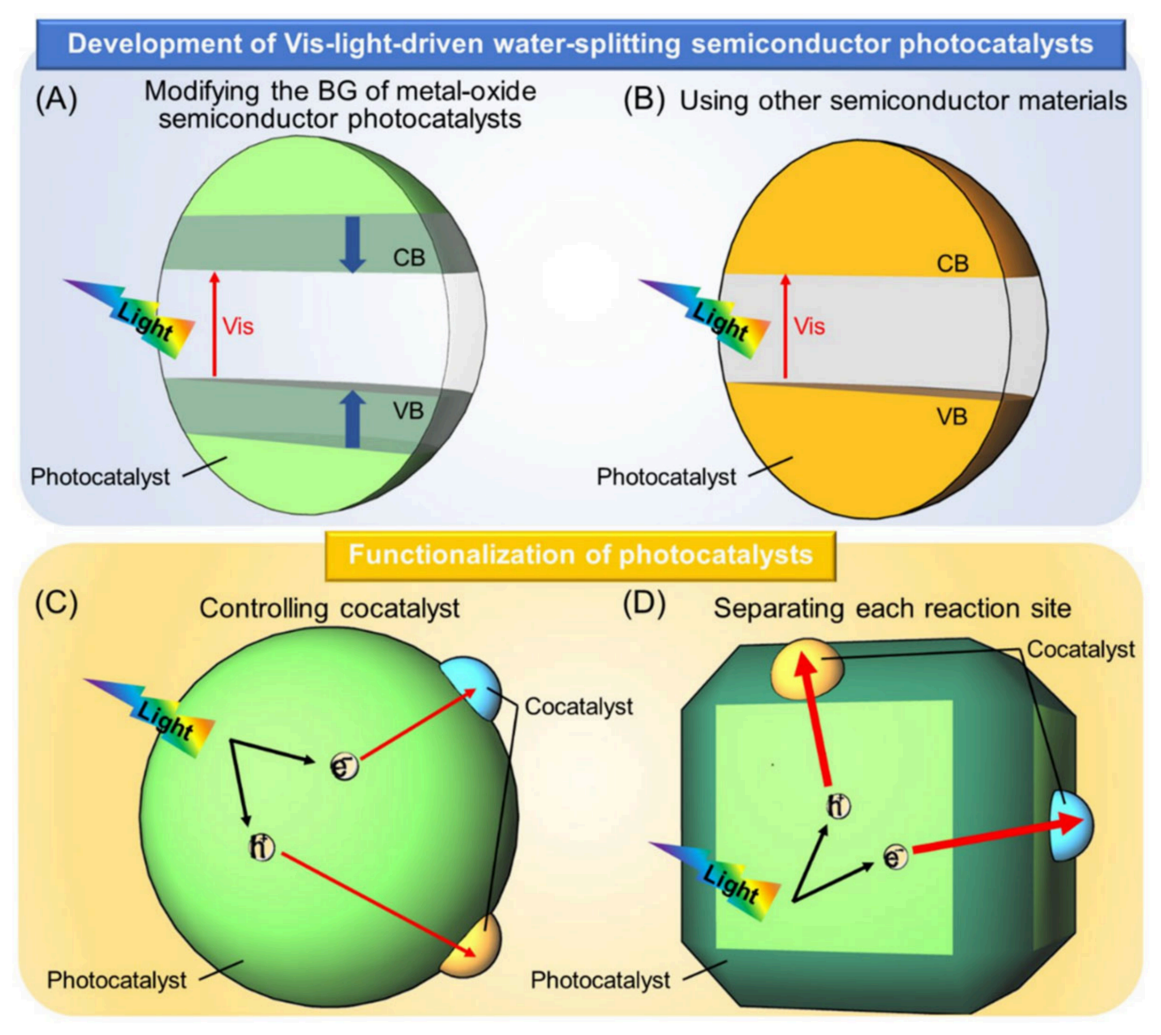
Overview of performance-enhancement strategies for MV_2_O_6_ photocatalysts, including morphology engineering, defect engineering, heterojunction construction, and cocatalyst loading, (open access) [36].

Photocatalytic performance of MV_2_O_6_ is strongly governed by crystal phase, morphology, defect concentration, and interfacial characteristics [34]. Conventional synthetic routes such as solid-state, hydrothermal, and sol–gel methods have yielded a variety of MV_2_O_6_ nanostructures, including nanorods, nanowires, and nanosheets. More recently, deep eutectic solvent (DES)-assisted synthesis has emerged as a greener and more controllable route, enabling phase-pure metavanadates with tailored crystal growth and intrinsic oxygen vacancies. In particular, DES-derived Zn/Cu metavanadates have been shown to contain reduced V^4+^ and metal cation states, together with oxygen-deficient structures that extend visible-light absorption and generate active sites for photocatalysis [8,35]. Figure 3 summarizes the primary performance-enhancement strategies for MV_2_O_6_ photocatalysts.

Defect engineering—particularly controlled generation of oxygen vacancies and V^4+^ states—improves visible-light absorption and charge separation [37,38]. Heterojunction construction (Type-II, Z-scheme, and S-scheme architectures) promotes spatial charge separation while preserving strong redox potentials [11,29]. The S-scheme configuration has gained prominence for overall water splitting applications. Additionally, cocatalyst loading (Pt, Au, Ni, CoO_x_, MoS_2_, RuO_2_) accelerates surface reactions by reducing overpotentials [39,40].

Despite these advances, several challenges still limit the practical deployment of MV_2_O_6_ photocatalysts, including thermodynamic restrictions arising from unfavorable conduction-band positions in some compositions, kinetic losses from bulk recombination, stability issues such as photocorrosion in Cu- and Ni-containing metavanadates, and the difficulty of achieving stoichiometric overall water splitting without sacrificial reagents. Overcoming these limitations will require a coordinated approach combining defect engineering, heterojunction design, cocatalyst optimization, and scalable synthesis. Accordingly, this review summarizes recent progress in MV_2_O_6_ photocatalysts, with emphasis on crystal structures, synthesis strategies—especially DES-based routes—modification approaches including Z-scheme and S-scheme heterojunctions, cocatalyst photodeposition, and remaining challenges toward industrially viable particulate photocatalysts [35,41,42]. Consequently, we extend our analysis to include PEC water oxidation studies, as they provide critical insights into charge-transport limitations and hole-transfer kinetics that are directly relevant to the oxygen-evolution half-reaction in particulate systems.

## 2. Crystal Structure and Electronic Properties of MV_2_O_6_ Metavanadates

The crystal structure and electronic properties of semiconductor photocatalysts play a decisive role in determining their light-harvesting capability, charge-carrier dynamics, and photocatalytic activity. Among vanadium-based semiconductors, divalent metal metavanadates (MV_2_O_6_; M = Zn, Ni, Co, Cu, Mn, Mg, and related cations) have attracted increasing attention because of their visible-light response, compositional tunability, and structural versatility. Unlike conventional binary oxides, MV_2_O_6_ compounds contain both transition-metal cations and vanadium-centered polyhedral units, enabling flexible modulation of electronic structure and charge transport pathways. These characteristics make MV_2_O_6_ materials promising candidates for photocatalytic and photoelectrochemical water-splitting applications.

### 2.1. Crystal Structure of MV_2_O_6_ Metavanadates

The crystal structures of MV_2_O_6_ compounds are strongly influenced by the ionic radius and electronic configuration of the divalent metal cation. Most members of the family crystallize in monoclinic structures composed of interconnected VO_6_ octahedra and MO_6_ polyhedra. The structural framework is generally built from edge-sharing or corner-sharing VO_6_ octahedral chains extending throughout the lattice, creating pathways for charge transport and influencing the optical and electronic properties of the material.

ZnV_2_O_6_, NiV_2_O_6_, CoV_2_O_6_, MnV_2_O_6_, and MgV_2_O_6_ are commonly reported in monoclinic crystal systems, although structural variations may occur depending on synthesis conditions and temperature. In contrast, CuV_2_O_6_ exhibits a lower-symmetry crystal structure arising from the Jahn–Teller distortion associated with Cu^2+^ ions, resulting in significant local lattice distortions and modified electronic interactions. These structural differences influence orbital overlap, carrier mobility, and band-edge positions, thereby affecting photocatalytic performance.

**Figure 4:**
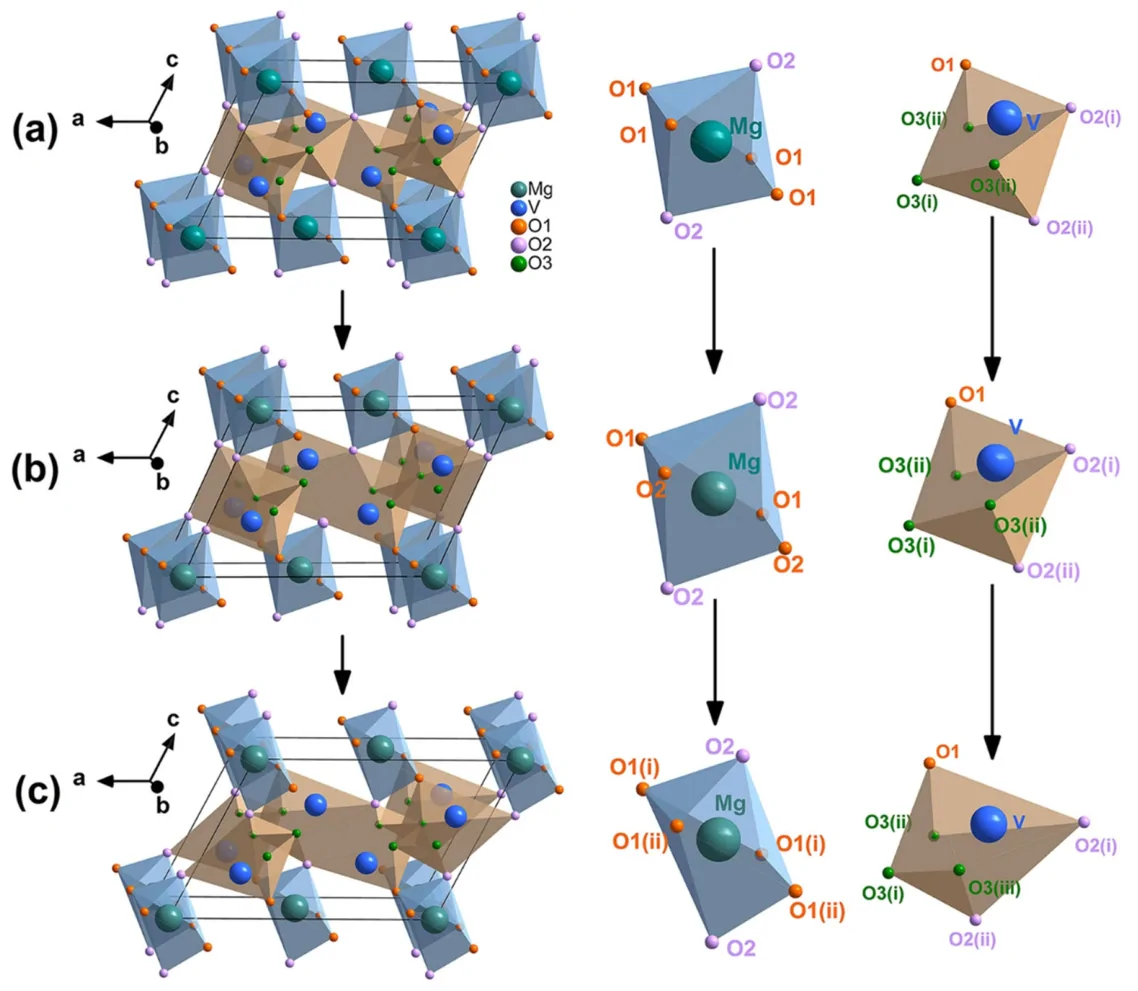
Pressure-induced structural evolution of the MV_2_O_6_ phase, showing the C2/m structure at 1.9 GPa (**a**) and 4.3 GPa (**b**), and the C2 structure at 27.4 GPa (**c**), (open access) [31].

To gain insight into the structural evolution of MV_2_O_6_ under compression, the optimized crystal structures at selected pressures are presented in Figure 4. The material initially crystallizes in the monoclinic C2/m phase at lower pressures and undergoes gradual lattice distortion with increasing pressure. At higher pressures, a structural phase transition occurs, resulting in the formation of the C2 phase. The corresponding atomic arrangements at representative pressures are illustrated to highlight the pressure-induced changes in coordination environment and crystal symmetry.

The metavanadate framework is dominated by V^5+^ ions in distorted VO_6_ octahedra. Since V^5+^ possesses a d^0^ electronic configuration, the empty V 3d orbitals play a central role in the formation of the conduction band. Variations in M–O bond lengths, V–O bond distances, and octahedral distortions directly affect the degree of orbital hybridization and electronic delocalization within the crystal lattice. Consequently, subtle structural modifications can significantly alter the optical and electronic properties of MV_2_O_6_ compounds. Table 1 summarizes the key crystallographic parameters for representative MV_2_O_6_ compounds.

An interesting example within the family is MgV_2_O_6_, which exhibits polymorphism and pressure-induced structural transformations. Thermodynamic investigations have revealed phase transitions between α- and β-MgV_2_O_6_, highlighting the structural flexibility of the metavanadate framework. Although MgV_2_O_6_ has received limited attention for photocatalytic applications, its structural stability and tunable lattice characteristics make it an attractive candidate for future investigations.

### 2.2. Electronic Structure and Optical Properties

The electronic structure of MV_2_O_6_ metavanadates originates primarily from the hybridization of O 2p and V 3d orbitals. Density functional theory (DFT) calculations and spectroscopic studies consistently indicate that the valence band is dominated by O 2p states, whereas the conduction band is largely derived from empty V 3d orbitals with varying contributions from the divalent metal cation. This electronic configuration facilitates ligand-to-metal charge transfer under visible-light irradiation and contributes to the favorable optical absorption characteristics of the metavanadate family [43].

One of the most attractive features of MV_2_O_6_ materials is their relatively narrow band-gap energy, typically ranging from approximately 1.8 to 2.5 eV. Such band gaps enable efficient utilization of visible light and compare favorably with traditional wide-band-gap photocatalysts such as TiO_2_. The exact band-gap value depends on the metal cation, crystal structure, synthesis route, and defect concentration. In general, CuV_2_O_6_ exhibits the narrowest reported band gap owing to strong Cu 3d–O 2p interactions, whereas MgV_2_O_6_ and ZnV_2_O_6_ tend to display slightly wider band gaps (Table 2).

In addition to optical absorption, the relative positions of the conduction and valence bands are crucial for photocatalytic water splitting. Available studies suggest that the band-edge positions of MV_2_O_6_ compounds vary considerably depending on composition and synthesis conditions. Consequently, caution should be exercised when comparing absolute band-edge values reported in different studies. Nevertheless, the overall trend indicates that several MV_2_O_6_ compounds possess valence-band positions sufficiently positive for water oxidation, whereas the driving force for proton reduction is often more composition-dependent. This explains why many reported MV_2_O_6_ systems exhibit stronger oxygen-evolution or photoanodic activity than hydrogen-evolution performance.

### 2.3. Charge Transport and Defect Chemistry

The photocatalytic performance of MV_2_O_6_ materials is not governed solely by band-gap energy but also by charge-carrier generation, transport, and recombination processes. Upon visible-light irradiation, electrons are excited from O 2p-derived valence-band states into V 3d-derived conduction-band states, generating electron–hole pairs capable of driving redox reactions. Efficient separation and migration of these carriers are essential for achieving high photocatalytic activity [48].

The interconnected VO_6_ octahedral framework provides pathways for charge transport and can promote electronic delocalization compared with isolated tetrahedral vanadate structures. However, similar to many oxide semiconductors, pristine MV_2_O_6_ compounds often suffer from rapid charge-carrier recombination, which significantly limits photocatalytic efficiency. Defect engineering has therefore emerged as an important strategy for improving charge separation and transport. Oxygen vacancies and mixed-valence species can introduce localized electronic states within the band gap, extend visible-light absorption, and facilitate carrier migration. Such defects may be generated during synthesis, thermal treatment, or reductive processing. Nevertheless, excessive defect concentrations can create recombination centers and reduce photocatalytic performance, emphasizing the need for precise defect control.

**Figure 5:**
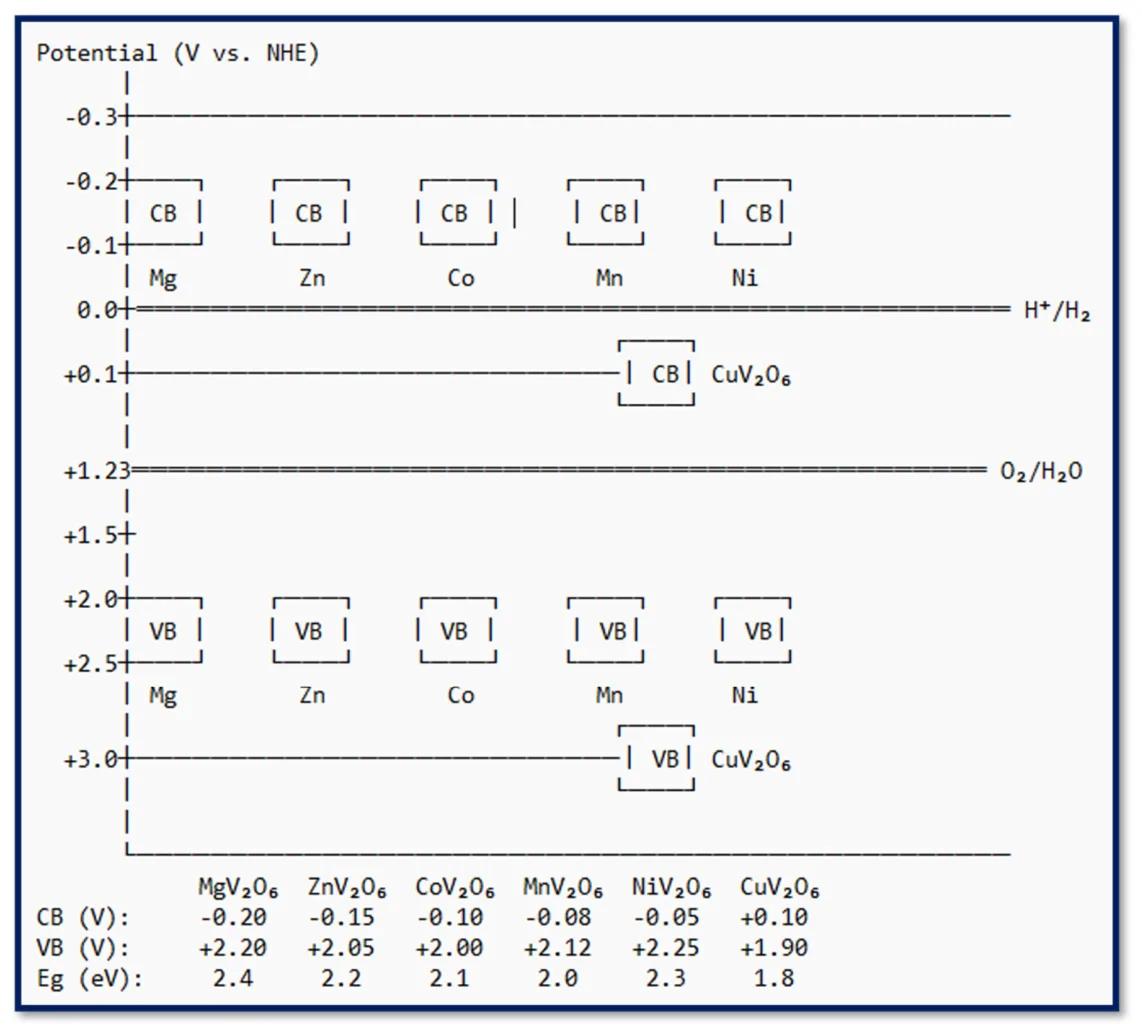
Band alignment diagram of MV_2_O_6_ metavanadates.

Overall, the electronic properties of MV_2_O_6_ metavanadates arise from the interplay between crystal structure, orbital hybridization, and defect chemistry. Their visible-light-responsive band gaps, tunable electronic structures, and adaptable crystal frameworks provide a strong foundation for photocatalytic water splitting. Understanding these structure–property relationships is essential for the rational design of advanced MV_2_O_6_ photocatalysts through morphology control, defect engineering, heterojunction construction, and cocatalyst integration. Figure 5 presents the band alignment diagram showing CB/VB edge positions of selected metavanadates relative to water redox potentials.

## 3. Synthesis and Micro/Nanostructure Engineering of MV_2_O_6_ Photocatalysts

The photocatalytic and photoelectrochemical performance of MV_2_O_6_ metavanadates is strongly influenced by their synthesis route, which determines crystal phase, morphology, particle size, defect concentration, surface area, and charge-transport characteristics. Unlike simple binary metal oxides, the synthesis of MV_2_O_6_ compounds often requires careful control of reaction conditions because multiple vanadium-containing phases, including orthovanadates and pyrovanadates, may coexist depending on precursor composition and thermal treatment. Consequently, the development of reliable synthesis strategies capable of controlling phase purity and microstructure is essential for optimizing photocatalytic performance.

### 3.1. Conventional Synthesis Routes

Solid-state synthesis remains the most widely employed method for preparing MV_2_O_6_ compounds. In this approach, stoichiometric amounts of metal oxide or metal salt precursors are mixed with vanadium sources and subsequently calcined at elevated temperatures to induce phase formation. This method has been successfully utilized for the preparation of ZnV_2_O_6_, NiV_2_O_6_, CuV_2_O_6_, MgV_2_O_6_, MnV_2_O_6_, and CoV_2_O_6_. The primary advantages of solid-state synthesis include simplicity, low cost, and scalability. However, the resulting materials generally possess relatively large particle sizes, low specific surface areas, and limited control over morphology, which may adversely affect photocatalytic activity.

To overcome these limitations, various wet-chemical methods have been developed. Hydrothermal and solvothermal techniques are among the most frequently reported approaches for synthesizing nanostructured MV_2_O_6_ materials. These methods enable controlled crystal growth under moderate temperatures and pressures, producing a wide range of morphologies, including nanorods, nanowires, nanosheets, and hierarchical architectures. The morphology can be tuned through precursor concentration, reaction temperature, solvent composition, pH, and the use of surfactants or structure-directing agents.

Sol–gel synthesis has also been employed to obtain homogeneous precursor mixtures and improved compositional control. The molecular-level mixing of metal ions facilitates phase formation at lower calcination temperatures and often results in smaller particle sizes compared with conventional solid-state routes. Nevertheless, post-calcination treatments are generally required to achieve sufficient crystallinity, which may lead to particle aggregation and reduced surface area.

Overall, hydrothermal, solvothermal, and sol–gel methods offer improved microstructural control compared with conventional solid-state synthesis and have therefore become important routes for preparing high-performance MV_2_O_6_ photocatalysts.

### 3.2. Emerging Solution-Based and Deep Eutectic Solvent Approaches

In recent years, alternative solution-based synthesis approaches have attracted increasing interest for the preparation of vanadate photocatalysts. Among these, deep eutectic solvents (DESs) have emerged as promising reaction media owing to their low volatility, low toxicity, tunable physicochemical properties, and ability to dissolve a wide range of inorganic precursors. DESs are typically formed through hydrogen-bond interactions between a hydrogen-bond donor and a hydrogen-bond acceptor, generating a liquid phase at temperatures significantly lower than those of the individual components. In vanadate synthesis, DESs can function simultaneously as solvents, reaction media, and structure-directing agents, promoting homogeneous precursor distribution and controlled nucleation.

Recent studies have demonstrated that DES-assisted synthesis can facilitate the formation of phase-pure vanadate materials under relatively mild conditions while offering improved control over particle morphology and defect generation. Furthermore, the unique chemical environment provided by DESs may promote the formation of oxygen vacancies and mixed-valence states, which can influence visible-light absorption and charge-transfer behavior, and surface catalytic activity. The reducing nature of certain DES components—particularly those containing hydroxyl or amine functional groups—can induce partial reduction of metal centers during synthesis, leading to the generation of oxygen vacancies that are charge-compensated by the formation of V^4+^ species. These oxygen vacancies introduce mid-gap states that extend the light absorption range and serve as electron-trapping sites that can suppress charge recombination, thereby enhancing photocatalytic performance.

A critical distinction between DES-assisted synthesis and conventional solid-state or hydrothermal methods lies in the concentration, type, and distribution of oxygen vacancies produced. Conventional high-temperature calcination typically generates oxygen vacancies primarily on the surface through thermal annealing, often accompanied by significant particle agglomeration and limited control over vacancy distribution. In contrast, the mild processing conditions and molecular-level homogeneity achievable in DES media enable more uniform incorporation of oxygen vacancies throughout the bulk of the material, as the solvent environment promotes intimate contact between precursors and facilitates controlled reduction during crystallization. Moreover, the hydrogen-bonding network of DESs can stabilize metastable vacancy configurations and prevent their annihilation during subsequent thermal treatments, resulting in higher overall vacancy concentrations compared to conventional methods. For instance, DES-synthesized BiVO_4_ has been reported to exhibit significantly higher V^4+^/V^5+^ ratios and enhanced photocatalytic activity relative to samples prepared by conventional hydrothermal routes, an effect attributed to the reductive environment provided by the DES. Similarly, ZnV_2_O_6_ synthesized in choline chloride–ethylene glycol DES has demonstrated improved visible-light absorption and photoelectrochemical performance, correlated with the presence of oxygen vacancies and associated V^4+^ states induced by the DES medium.

Despite these promising demonstrations, the application of DES-assisted synthesis specifically to the MV_2_O_6_ family remains at an early stage, and systematic comparative studies quantifying vacancy concentrations across different synthesis methods are currently limited. Initial evidence suggests that the type of hydrogen-bond donor and acceptor, the precursor concentration, and the synthesis temperature all play crucial roles in determining the nature and extent of oxygen vacancy formation. For example, DESs containing reducing components such as urea or ethylene glycol tend to generate higher concentrations of V^4+^ species than those based solely on choline chloride, while synthesis temperatures above 150 °C may promote partial reoxidation and vacancy annihilation. These observations underscore the importance of careful optimization of DES composition and processing parameters to achieve the desired defect chemistry.

Looking forward, DES-assisted synthesis represents a promising and scalable route for producing nanostructured MV_2_O_6_ photocatalysts with tailored oxygen vacancy concentrations, controlled morphology, and enhanced visible-light responsiveness. Nevertheless, further systematic investigations—including quantitative comparisons of oxygen vacancy densities, electron paramagnetic resonance (EPR) spectroscopy, and X-ray photoelectron spectroscopy (XPS) analysis—are needed to fully elucidate the relationship between DES synthesis parameters, defect structures, and photocatalytic performance in MV_2_O_6_ systems. Such studies will be essential for establishing design principles that enable the rational engineering of oxygen vacancies in metavanadate photocatalysts for improved solar hydrogen production.

#### Comparative Assessment of Synthesis Methods

The selection of an appropriate synthesis route plays a decisive role in determining the structural and photocatalytic properties of MV_2_O_6_ materials. Each synthesis method possesses distinct advantages and limitations that directly influence crystal quality, morphology, defect chemistry, scalability, and production cost.

Conventional solid-state synthesis remains attractive because of its operational simplicity, low cost, high product yield, and suitability for large-scale production. However, the high calcination temperatures typically required often produce large particles with low specific surface areas, significant agglomeration, and limited control over morphology and defect distribution, thereby restricting photocatalytic performance.

In contrast, hydrothermal and solvothermal methods provide excellent control over crystal growth and enable the synthesis of well-defined nanostructures, including nanorods, nanosheets, nanowires, and hierarchical architectures. These methods generally produce materials with higher crystallinity, larger surface areas, and improved charge-transport properties compared with solid-state synthesis. Nevertheless, they require specialized pressure-resistant autoclaves, relatively long reaction times, and may present challenges for large-scale industrial production.

Sol–gel synthesis offers homogeneous mixing of precursor species at the molecular level, allowing improved compositional uniformity and phase formation at relatively lower calcination temperatures. Consequently, smaller particle sizes and better chemical homogeneity can often be achieved. However, subsequent heat treatment is usually necessary to obtain sufficient crystallinity, which may induce particle aggregation and reduce the accessible surface area. Furthermore, residual organic species and process complexity may require careful optimization.

Among emerging approaches, deep eutectic solvent (DES)-assisted synthesis has attracted increasing attention because it combines environmentally friendly processing with excellent control over nucleation, crystal growth, morphology, and defect formation. The unique hydrogen-bonding environment of DESs facilitates homogeneous precursor distribution and promotes the controlled generation of oxygen vacancies and mixed-valence V^4+^ species, thereby enhancing visible-light absorption, charge separation, and photocatalytic activity. DES-based synthesis also operates under relatively mild conditions with low volatility and reduced toxicity. Despite these advantages, the application of DESs to the MV_2_O_6_ family remains in its infancy, and systematic optimization of solvent composition, reaction parameters, and scalability is still required.

Overall, no single synthesis method is universally superior for all MV_2_O_6_ compositions. Solid-state synthesis is preferable for economical large-scale production, whereas hydrothermal, solvothermal, and sol–gel methods provide superior microstructural control for high-performance photocatalysts. DES-assisted synthesis represents one of the most promising next-generation strategies by simultaneously enabling morphology engineering and defect modulation under environmentally benign conditions. Future studies integrating these synthesis approaches with advanced defect engineering and heterojunction design are expected to further enhance the photocatalytic efficiency of MV_2_O_6_ materials.

### 3.3. Morphology-Controlled Synthesis

Morphology engineering has become an important strategy for improving the photocatalytic performance of MV_2_O_6_ materials. The morphology of a photocatalyst directly affects light harvesting, surface reaction kinetics, charge-carrier transport, and the availability of active sites. One-dimensional nanostructures, such as nanorods and nanowires, facilitate directional charge transport and reduce carrier recombination by shortening diffusion pathways. Two-dimensional nanosheets provide large exposed surface areas and abundant reactive sites, which are advantageous for photocatalytic reactions. Meanwhile, three-dimensional hierarchical structures, including nanoflowers, microspheres, and porous architectures, combine high surface area with enhanced light scattering and multiple reflection effects, leading to improved photon utilization.

Various morphologies have been reported for MV_2_O_6_ compounds depending on the synthesis method employed. Hydrothermal and solvothermal routes commonly yield rod-like, wire-like, sheet-like, and flower-like structures, whereas template-assisted and electrospinning techniques can produce porous architectures and one-dimensional fibers. The growing ability to precisely control morphology offers significant opportunities for optimizing the photocatalytic performance of MV_2_O_6_-based materials. Table 3 summarizes morphology control strategies for different MV_2_O_6_ compositions.

Among the MV_2_O_6_ compounds, ZnV_2_O_6_, NiV_2_O_6_, and MnV_2_O_6_ have received the greatest attention for morphology-controlled synthesis, whereas MgV_2_O_6_ remains largely unexplored. The development of nanostructured MgV_2_O_6_ and its integration into heterojunction photocatalysts may therefore represent an important future research direction.

Although reports on morphology-controlled synthesis and photocatalytic water splitting are currently unavailable, the structural similarity of MgV_2_O_6_ to other MV_2_O_6_ compounds suggests that nanostructuring, defect engineering, and heterojunction construction could significantly improve its photocatalytic potential. Therefore, MgV_2_O_6_ represents an attractive yet largely unexplored platform for future investigations aimed at understanding composition–structure–activity relationships within the MV_2_O_6_ family.

### 3.4. Defect Engineering via Synthesis Control

Defect engineering is increasingly recognized as an effective strategy for enhancing the photocatalytic activity of metal vanadates. Structural defects, particularly oxygen vacancies and mixed-valence metal centers, can modify electronic structures, improve visible-light absorption, and facilitate charge-carrier separation.

In MV_2_O_6_ systems, oxygen vacancies may be introduced through controlled synthesis conditions, thermal treatments, reducing atmospheres, or solution-based synthesis approaches. These defects can create localized electronic states within the band gap, extending light absorption into the visible region and enhancing charge-transfer processes. However, excessive defect concentrations may act as recombination centers, highlighting the importance of optimizing defect density. The relationship between synthesis conditions, defect chemistry, and photocatalytic activity remains insufficiently understood for many MV_2_O_6_ compositions. Future studies combining advanced spectroscopic characterization with theoretical calculations are therefore required to establish clear structure–property relationships and guide the rational design of defect-engineered MV_2_O_6_ photocatalysts.

Overall, advances in synthesis methodologies, morphology control, and defect engineering have significantly expanded the opportunities for tailoring the physicochemical properties of MV_2_O_6_ materials. Continued progress in these areas will be essential for realizing the full potential of MV_2_O_6_ photocatalysts in solar-driven hydrogen production and water-splitting applications.

## 4. Heterojunction and Cocatalyst Engineering for Enhanced Water Splitting

Although MV_2_O_6_ metavanadates exhibit favorable visible-light absorption, tunable electronic structures, and good chemical stability, their photocatalytic performance remains limited by rapid charge-carrier recombination, insufficient charge-transfer efficiency, and sluggish surface reaction kinetics. Furthermore, several members of the MV_2_O_6_ family have not yet demonstrated efficient overall water splitting, despite possessing suitable optical band gaps. Consequently, the development of heterojunction architectures and cocatalyst-modified systems has emerged as an effective strategy for enhancing performance in both photocatalytic (particulate suspension) and photoelectrochemical (PEC) configurations. It is important to note, however, that while these strategies are conceptually applicable to both systems, their implementation and evaluation differ substantially—PEC systems benefit from external bias and electrolyte effects that can artificially enhance charge separation, whereas particulate systems rely solely on internal electric fields and interfacial contacts. Throughout this section, we discuss these engineering approaches with explicit attention to their relevance and demonstrated efficacy in each configuration.

Heterojunction construction promotes the spatial separation of photogenerated electrons and holes through interfacial charge transfer, thereby suppressing recombination losses and improving photocatalytic efficiency. Likewise, cocatalyst loading provides active surface sites for hydrogen evolution reaction (HER) and oxygen evolution reaction (OER), facilitating charge extraction and accelerating interfacial reaction kinetics. Together, these approaches have become central to the design of high-performance vanadate-based photocatalysts and are expected to play a critical role in the future development of MV_2_O_6_ systems [58].

### 4.1. Heterojunction Strategies

Heterojunction engineering involves coupling two semiconductors with complementary electronic structures to generate an interfacial electric field that promotes directional charge migration. Such interfaces improve charge separation, extend visible-light utilization, and enhance photocatalytic activity. Based on the charge-transfer mechanism, heterojunctions can generally be classified into Type-II, Z-scheme, and S-scheme architectures [59]. While the fundamental principles of these junctions are universal, their practical performance in particulate systems depends critically on achieving intimate solid–solid contact and maintaining favorable band alignment without the assistance of external bias, making interface engineering particularly crucial for slurry-based photocatalysis.

#### 4.1.1. Type-II Heterojunctions

Type-II heterojunctions are characterized by staggered band alignment between two semiconductors. Upon illumination, photogenerated electrons migrate from the semiconductor with the more negative conduction band to the one with the less negative conduction band, while holes move in the opposite direction. This spatial separation effectively suppresses electron–hole recombination and prolongs carrier lifetimes.

**Figure 6:**
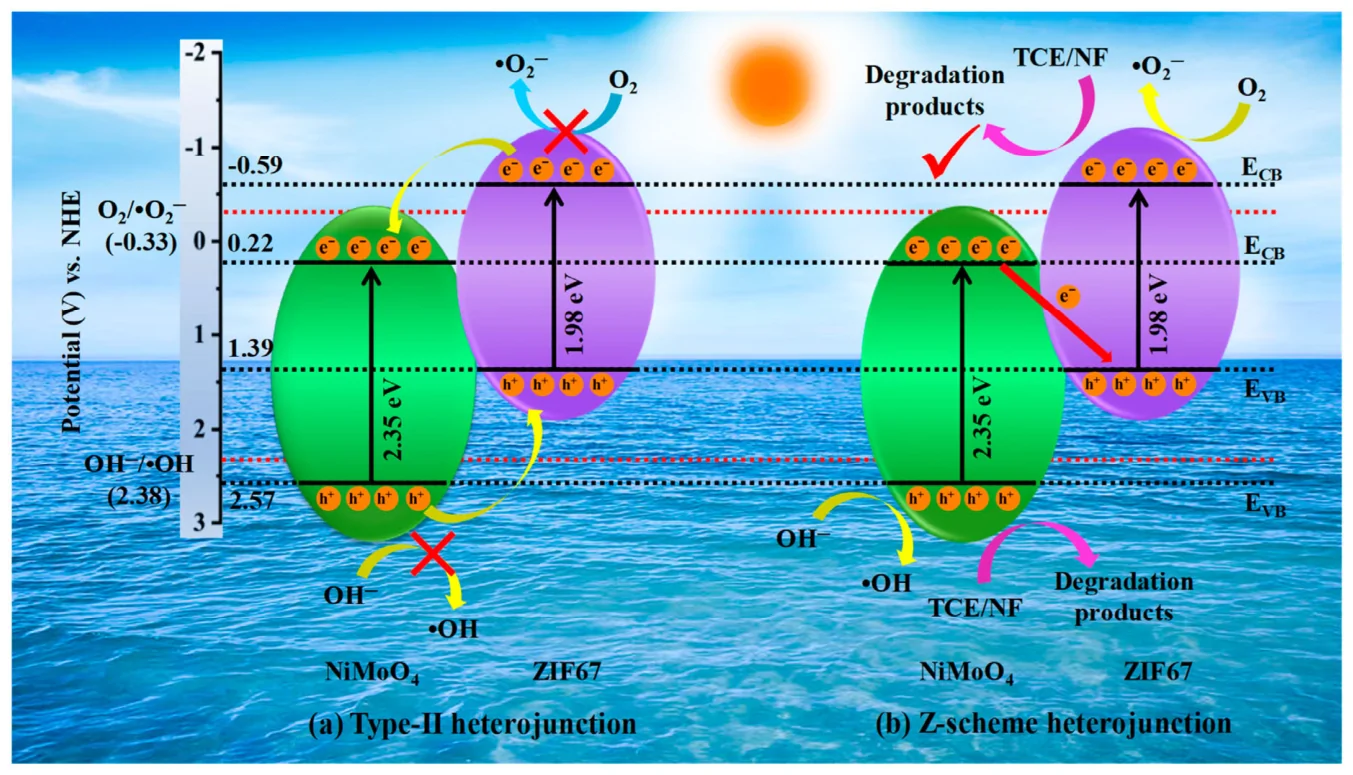
Schematic comparison of (**a**) Type-II and (**b**) Z-scheme charge transfer mechanisms, showing band alignment and carrier migration pathways (open access) [60].

Although Type-II heterojunctions have been extensively investigated in various vanadate systems, only limited reports are available for MV_2_O_6_-based photocatalysts. ZnV_2_O_6_ has attracted particular interest because of its suitable visible-light absorption and favorable electronic structure. Several studies have demonstrated that coupling ZnV_2_O_6_ with wide-band-gap semiconductors or conductive supports improves charge separation and photocurrent generation. Similar strategies have been successfully employed in CeVO_4_-, BiVO_4_-, and GdVO_4_-based photocatalysts, highlighting the potential of Type-II architectures for enhancing the performance of MV_2_O_6_ materials. Figure 6 presents a schematic comparison of Type-II and Z-scheme charge transfer mechanisms. As shown in Figure 6a, Type-II heterojunctions achieve spatial separation but at the cost of reduced redox potentials because electrons accumulate at a less negative CB and holes accumulate at a less positive VB.

Despite their effectiveness in charge separation, Type-II heterojunctions often suffer from weakened redox capability because the accumulated electrons and holes reside in less energetic conduction and valence bands, respectively. Consequently, their application in overall water splitting remains somewhat limited compared with more advanced heterojunction configurations, particularly for particulate systems where the absence of external bias makes it challenging to drive thermodynamically demanding reactions.

#### 4.1.2. Z-Scheme Heterojunctions

Z-scheme heterojunctions have emerged as one of the most effective approaches for photocatalytic water splitting because they simultaneously achieve efficient charge separation and strong redox capability. Inspired by natural photosynthesis, Z-scheme systems promote selective recombination of low-energy electrons and holes at the interface, preserving highly reducing electrons and highly oxidizing holes on opposite semiconductors. This mechanism is especially advantageous for particulate photocatalysis, where maintaining high redox potentials without external assistance is essential for driving both half-reactions simultaneously.

For MV_2_O_6_ materials, Z-scheme design is particularly attractive because it can compensate for unfavorable band-edge positions and enhance overall water-splitting performance. For example, CuV_2_O_6_ possesses excellent visible-light absorption but limited reduction capability. Coupling CuV_2_O_6_ with a reduction photocatalyst such as g-C_3_N_4_, CdS, or other narrow-band-gap semiconductors could theoretically preserve the strong reduction potential of the partner semiconductor while utilizing the oxidation capability of CuV_2_O_6_. Similarly, ZnV_2_O_6_ and NiV_2_O_6_ may benefit from Z-scheme architectures that maintain high redox potentials while promoting efficient charge separation. As illustrated in Figure 6b, this mechanism not only ensures efficient charge separation but also maintains high redox potentials, making Z-scheme systems highly effective for challenging photocatalytic transformations such as water splitting.

Although experimental reports on MV_2_O_6_-based Z-scheme heterojunctions remain limited, recent studies on related vanadate systems—including BiVO_4_ and InVO_4_—have demonstrated substantial improvements in hydrogen evolution, oxygen evolution, and pollutant degradation activities. These findings suggest that Z-scheme engineering represents one of the most promising directions for future MV_2_O_6_ photocatalyst development, with particular potential for enabling overall water splitting in particulate systems where both reduction and oxidation sites must be simultaneously active.

However, the application of Z-scheme architectures to MV_2_O_6_ materials presents several specific challenges that warrant careful consideration. First, the relatively positive conduction band positions of many MV_2_O_6_ members, particularly NiV_2_O_6_ and CuV_2_O_6_, require exceptionally careful selection of partner semiconductors to ensure effective band alignment while maintaining sufficient redox potentials. Second, the formation of intimate, defect-free interfaces between MV_2_O_6_ and partner materials is complicated by differences in lattice parameters and crystal structures, which can introduce interfacial defects that act as recombination centers. Third, the stability of certain MV_2_O_6_ compounds under operating conditions—particularly those containing redox-active transition metals—remains a concern, as photocorrosion could compromise long-term performance. Finally, the limited mechanistic understanding of charge transfer processes in MV_2_O_6_-based heterojunctions, due to the scarcity of advanced spectroscopic investigations, hinders the rational design of optimized systems. Addressing these challenges through systematic interface engineering, advanced characterization, and careful material selection will be essential for realizing the full potential of Z-scheme architectures in MV_2_O_6_ photocatalysis.

#### 4.1.3. Step-Scheme (S-Scheme) Heterojunctions

The step-scheme (S-scheme) heterojunction is an emerging photocatalytic architecture that integrates the efficient charge separation characteristics of Type-II heterojunctions with the strong redox capability of direct Z-scheme systems. Unlike conventional Type-II heterojunctions, where both electrons and holes migrate to lower-energy bands at the expense of redox potential, the S-scheme configuration selectively preserves the charge carriers with the highest reduction and oxidation abilities, thereby maximizing photocatalytic efficiency.

The formation of an S-scheme heterojunction begins with the contact between a reduction photocatalyst (RP), which possesses a higher Fermi level, and an oxidation photocatalyst (OP), having a lower Fermi level. Upon contact, electrons spontaneously transfer from the RP to the OP until Fermi-level equilibrium is established. This electron redistribution produces band bending at the interface and generates a built-in internal electric field directed from the RP toward the OP. The combined effects of the internal electric field and band bending play a crucial role in directing charge migration after light irradiation [61].

**Figure 7:**
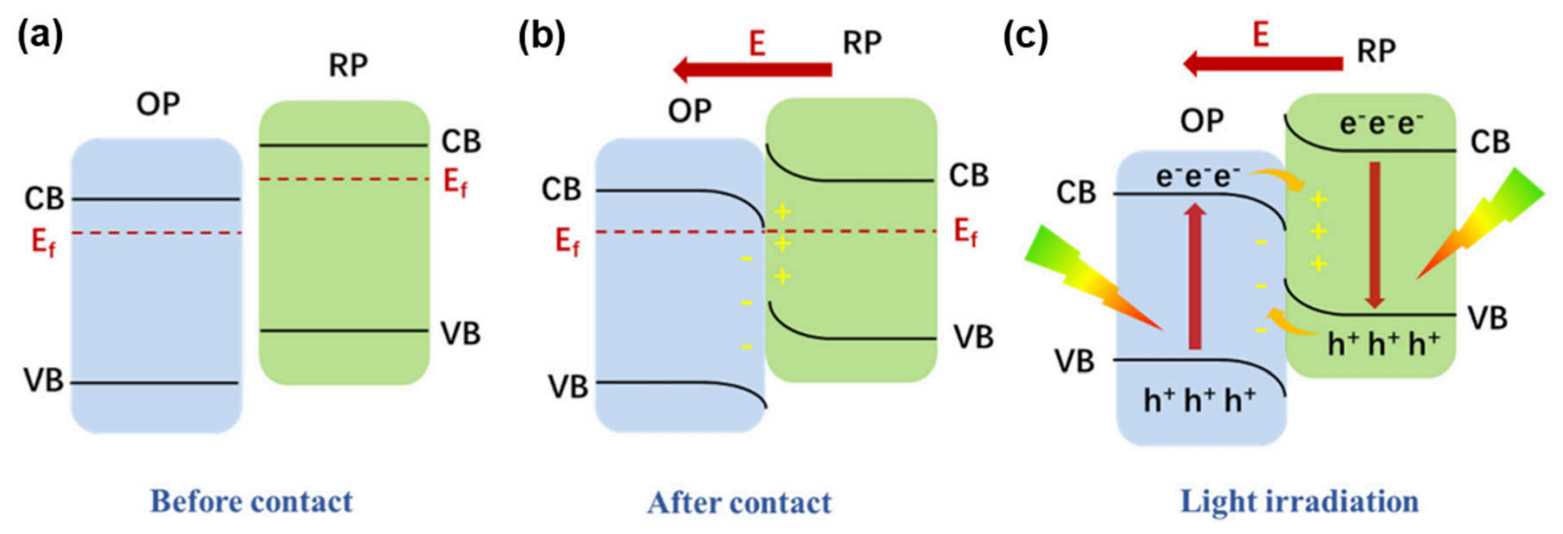
The energy band diagram of an S-scheme heterojunction, illustrating the formation of the internal electric field and charge transfer pathway; (**a**) before contact; (**b**) after contact; and (**c**) photogenerated carrier transfer under light irradiation (open access) [62].

Under illumination, both semiconductors generate electron–hole pairs. The photogenerated electrons in the conduction band of the oxidation photocatalyst preferentially recombine with the holes in the valence band of the reduction photocatalyst at the interface under the influence of the built-in electric field. Consequently, only the highly energetic electrons in the conduction band of the reduction photocatalyst and the strongly oxidizing holes in the valence band of the oxidation photocatalyst remain available to participate in photocatalytic reactions. This selective recombination effectively suppresses electron–hole recombination while simultaneously preserving the strong reduction and oxidation potentials required for water splitting (Figure 7).

Compared with conventional Type-II systems, S-scheme heterojunctions preserve strong redox ability while maintaining efficient charge separation. Furthermore, the built-in electric field facilitates directional carrier migration and suppresses undesirable back reactions. Although reports involving MV_2_O_6_-based S-scheme photocatalysts are currently scarce, this strategy offers significant potential for future development of efficient water-splitting systems based on ZnV_2_O_6_, NiV_2_O_6_, and CuV_2_O_6_.

The limited experimental validation of S-scheme heterojunctions in MV_2_O_6_ systems can be attributed to several fundamental challenges. First, the construction of S-scheme heterojunctions requires precise matching of work functions (Φ) between the reduction photocatalyst and the oxidation photocatalyst, specifically that Φ_RP < Φ_OP. For MV_2_O_6_ compounds, the work function values are not yet well established, making the rational selection of suitable partner semiconductors difficult. Second, the formation of the internal electric field essential for S-scheme charge transfer depends critically on the quality of the interfacial contact. Achieving such contact with MV_2_O_6_ materials is challenging due to their diverse crystal structures and surface chemistries, which vary significantly depending on synthesis conditions. Third, the metastable nature of some MV_2_O_6_ polymorphs may complicate the controlled synthesis of S-scheme heterojunctions, as the high-temperature processing often required for heterojunction formation could induce undesirable phase transformations. Finally, the lack of systematic studies employing advanced characterization techniques—such as Kelvin probe force microscopy, surface photovoltage spectroscopy, and transient absorption spectroscopy—limits the mechanistic understanding needed to guide the design of efficient S-scheme systems. Future research should prioritize these fundamental investigations to establish design principles for MV_2_O_6_-based S-scheme heterojunctions.

Overall, the application of Type-II, Z-scheme, and S-scheme heterojunctions provides an effective route to overcome the intrinsic limitations of MV_2_O_6_ photocatalysts. However, it is important to recognize that the limited availability of experimental reports on MV_2_O_6_-based heterojunctions—particularly for Z-scheme and S-scheme architectures—reflects the significant challenges inherent to this material family. These challenges include stringent band alignment requirements, difficulties in achieving intimate interfacial contact, concerns regarding material stability and photocorrosion, and an incomplete mechanistic understanding of charge transfer processes. Future studies should focus on interface engineering, band-structure matching, and mechanistic investigations to establish design principles for high-performance heterostructures. Critically, while PEC studies have validated the efficacy of these approaches for individual half-reactions, their successful translation to particulate overall water splitting will require careful optimization of interfacial contacts, precise control over junction formation, and validation under suspension conditions without external bias. Systematic investigations employing advanced characterization techniques will be essential to overcome these challenges and unlock the full potential of heterojunction engineering for MV_2_O_6_ photocatalysis.

### 4.2. Cocatalyst Engineering

**Figure 8:**
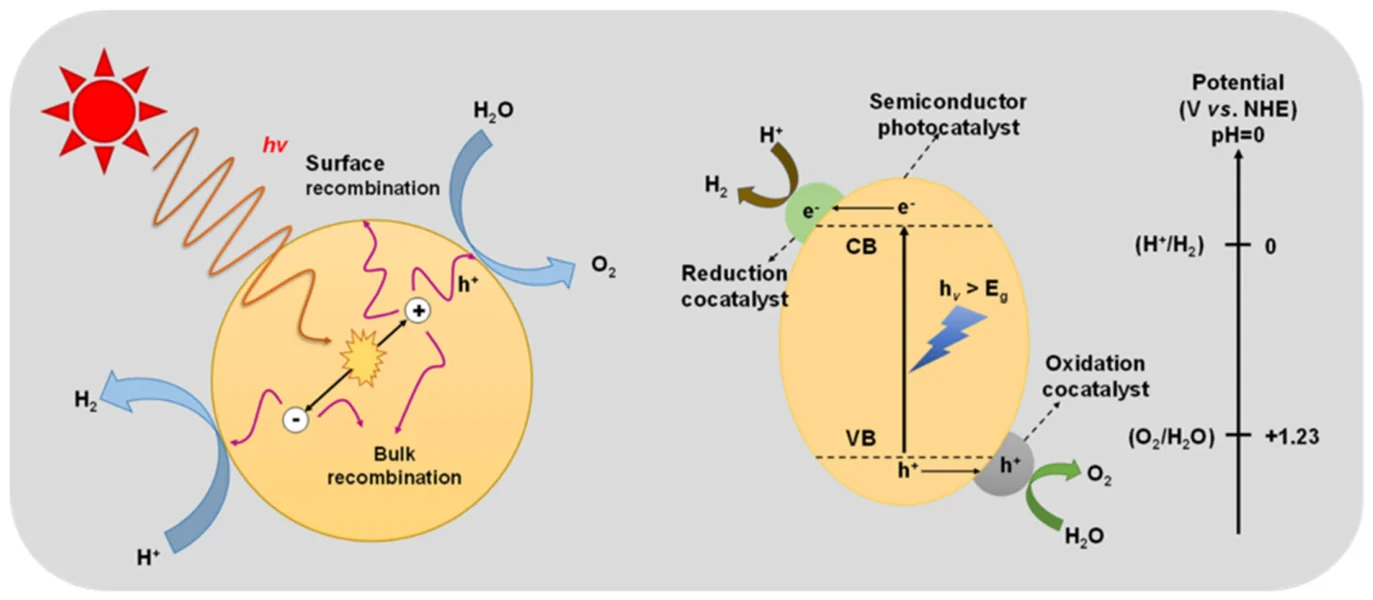
Schematic illustration of photocatalytic overall water splitting, (open access) [63].

In addition to heterojunction construction, cocatalyst loading is one of the most effective approaches for enhancing photocatalytic water splitting. Cocatalysts improve charge separation by acting as electron or hole sinks, lower activation barriers for surface reactions, and provide active catalytic sites for hydrogen and oxygen evolution. Moreover, they can suppress photocorrosion and improve long-term photocatalytic stability (Figure 8). It is worth emphasizing that cocatalyst functionality is inherently system-dependent: in PEC configurations, cocatalysts primarily facilitate charge extraction at the semiconductor–electrolyte interface under applied bias, whereas in particulate photocatalysis, they must also compensate for the absence of external bias by providing highly efficient catalytic sites that minimize overpotentials and drive surface reactions spontaneously.

#### 4.2.1. Cocatalysts for Hydrogen Evolution

Hydrogen-evolution cocatalysts facilitate proton reduction by providing highly active electron-accepting sites. Noble metals such as Pt, Pd, and Au remain the most efficient HER cocatalysts owing to their excellent conductivity and near-optimal hydrogen adsorption energies. Photodeposition is widely employed to deposit these metals onto semiconductor surfaces, enabling intimate contact between the cocatalyst and photocatalyst. In particulate systems, the uniform dispersion of cocatalyst nanoparticles is particularly critical to ensure that photogenerated electrons are efficiently harvested before recombination occurs.

Although systematic cocatalyst studies on MV_2_O_6_ materials remain limited, the successful application of Pt and other noble metals in vanadate-based photocatalysts suggests that similar approaches could significantly enhance the hydrogen-evolution performance of ZnV_2_O_6_ and MnV_2_O_6_. To reduce cost and improve scalability, increasing attention is being directed toward earth-abundant alternatives such as Ni_2_P, CoP, MoS_2_, transition-metal sulfides, and metal phosphides. For particulate photocatalysis, the development of robust earth-abundant cocatalysts is especially important, as their integration must achieve performance comparable to noble metals while maintaining long-term stability in suspension.

#### 4.2.2. Cocatalysts for Oxygen Evolution

The oxygen evolution reaction is kinetically more demanding than hydrogen evolution because it involves multiple proton-coupled electron-transfer steps and O–O bond formation. Therefore, efficient oxygen-evolution cocatalysts are essential for achieving overall water splitting.

Transition-metal oxides and oxyhydroxides, including CoO_x_, NiO_x_, FeOOH, RuO_2_, and IrO_2_, are commonly employed as OER cocatalysts. For photoanode systems such as ZnV_2_O_6_ and NiV_2_O_6_, these cocatalysts facilitate hole extraction, reduce interfacial charge recombination, and accelerate water oxidation kinetics. Among them, cobalt- and nickel-based cocatalysts are particularly attractive because of their abundance, stability, and high OER activity under alkaline conditions. However, it should be noted that the impressive OER performance observed in PEC configurations does not automatically translate to particulate systems, where cocatalysts must function without the assistance of external bias and in the presence of both reduction and oxidation sites on the same particle.

In future MV_2_O_6_ photocatalytic systems, the rational integration of HER and OER cocatalysts with optimized heterojunction architectures is expected to be a key strategy for simultaneously improving charge separation, reaction kinetics, and overall solar-to-hydrogen conversion efficiency.

## 5. Photocatalytic Performance of MV_2_O_6_-Based Systems

### 5.1. Hydrogen Evolution Performance

The photocatalytic and photoelectrochemical properties of divalent metal vanadates (MV_2_O_6_, M = Zn, Ni, Cu, Mg, Mn, and Co) have attracted increasing attention owing to their narrow band gaps, visible-light absorption capabilities, and tunable electronic structures. However, compared with extensively investigated vanadates such as BiVO_4_, InVO_4_, and AgVO_3_, studies focusing on solar hydrogen production over MV_2_O_6_ materials remain relatively scarce. Existing research has primarily concentrated on photocatalytic pollutant degradation, photoelectrochemical (PEC) water oxidation, and electronic structure investigations, with only a few reports demonstrating direct photocatalytic hydrogen evolution. It is important to recognize that while PEC measurements provide valuable insights into intrinsic material properties and charge-transport limitations, they benefit from external bias that actively drives charge separation—a condition not present in particulate photocatalysis. Consequently, PEC performance should be interpreted as an indicator of potential rather than direct evidence of photocatalytic activity.

**Figure 9:**
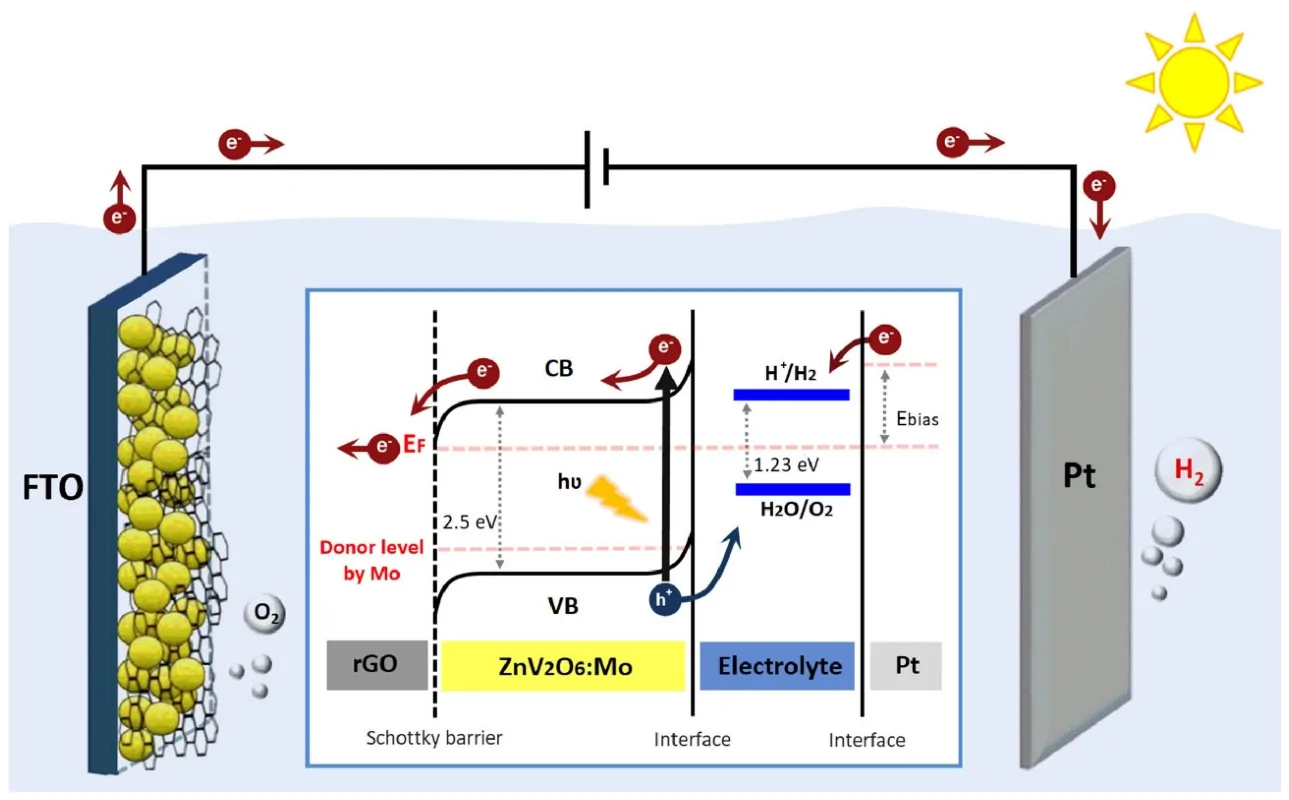
Schematic illustration of the charge-transfer process in the ZnV_2_O_6_:Mo/rGO photoanode during PEC water-splitting reactions, Reprinted with permission from Elsevier 2021 [64].

Among the reported systems, ZnV_2_O_6_ has emerged as a promising visible-light-responsive semiconductor due to its moderate band gap (2.2–2.5 eV) and suitable conduction-band potential for proton reduction. Although direct photocatalytic hydrogen evolution studies remain limited, ZnV_2_O_6_ has shown encouraging performance as a photoanode for PEC water splitting. In particular, Mo-doped ZnV_2_O_6_/reduced graphene oxide (rGO) photoanodes exhibited significantly enhanced photocurrent density (2.07 mA cm^−2^ at 1.23 V vs. RHE) and an incident photon-to-current conversion efficiency (IPCE) of 17% at 370 nm. The improved performance was attributed to increased charge-carrier concentration induced by Mo doping and efficient electron transport through the graphene network, which collectively promoted charge separation and interfacial transfer processes. These findings indicate that ZnV_2_O_6_-based heterostructures are promising candidates for solar hydrogen generation through PEC pathways (Figure 9).

NiV_2_O_6_ has been studied predominantly as a visible-light-active photoanode rather than a hydrogen-evolving photocatalyst. Dang et al. demonstrated that triclinic NiV_2_O_6_ thin films possess indirect and direct optical band gaps of approximately 2.1 and 2.4 eV, respectively, and exhibit stable photoelectrochemical water oxidation under alkaline conditions. Notably, approximately 45% of the generated photocurrent originated from wavelengths longer than 420 nm, confirming efficient visible-light utilization. Oxygen evolution measurements further verified the capability of NiV_2_O_6_ for water oxidation. However, the relatively positive conduction-band position limits its proton-reduction ability, explaining the scarcity of photocatalytic hydrogen-evolution reports for this material.

CuV_2_O_6_ possesses strong visible-light absorption due to Cu–O–V charge-transfer transitions and has been investigated for photocatalysis, photoelectrochemistry, and thermochemical hydrogen-production processes. Nevertheless, direct photocatalytic hydrogen evolution over CuV_2_O_6_ remains largely unexplored. The limited activity is generally attributed to rapid electron–hole recombination and insufficient reducing power of the conduction band. Consequently, current research mainly focuses on employing CuV_2_O_6_ as a heterojunction component or catalytic material rather than as a standalone photocatalyst for hydrogen evolution.

MgV_2_O_6_ is among the least explored members of the MV_2_O_6_ family with respect to solar fuel production. Available studies are largely confined to crystal-structure analysis, optical characterization, dielectric properties, and general photocatalytic degradation applications. To date, no significant reports demonstrating either photocatalytic or photoelectrochemical hydrogen production over MgV_2_O_6_ have been identified. Its relatively wider band gap and poor electrical conductivity may contribute to the lack of hydrogen-production activity. Therefore, MgV_2_O_6_ remains an open research area for future photocatalytic investigations, with initial efforts needed to establish its fundamental photoelectrochemical properties.

**Figure 10:**
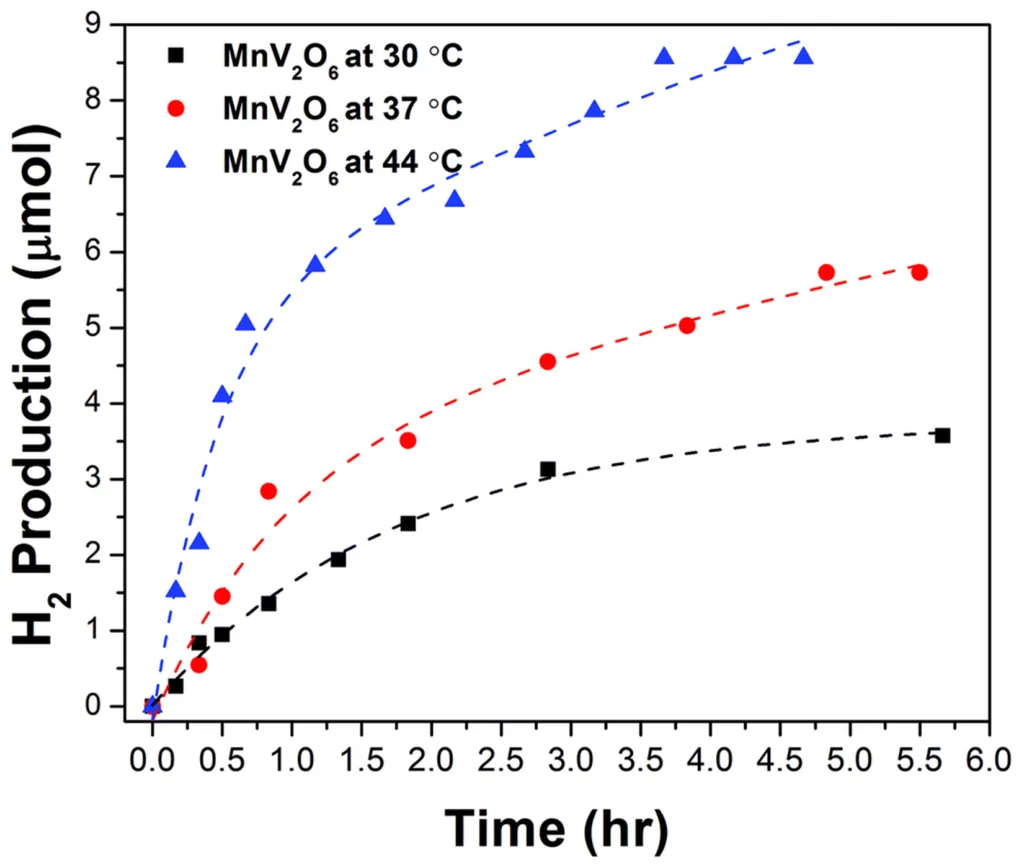
Temperature-dependent hydrogen production by MnV_2_O_6_ in 20% methanol solution under visible-light irradiation, Reprinted with permission from RSC 2017 [65].

Among all MV_2_O_6_ compounds, MnV_2_O_6_ exhibits the most convincing evidence for direct photocatalytic hydrogen production. Zoellner et al. reported that p-type MnV_2_O_6_ possesses favorable conduction- and valence-band positions of −0.464 V and +0.985 V versus RHE, respectively, enabling both hydrogen and oxygen evolution under visible-light irradiation. Suspended-particle photocatalytic experiments confirmed the generation of H_2_ and O_2_, while the hydrogen-evolution rate increased substantially with increasing reaction temperature (Figure 10). The enhanced photocatalytic activity was attributed to charge-transfer excitation from Mn 3d orbitals to V 3d orbitals, favorable band-edge alignment, and efficient charge separation associated with its p-type semiconducting nature. Notably, MnV_2_O_6_ remains one of the very few single-phase MV_2_O_6_ materials reported to directly evolve hydrogen under visible light.

**Figure 11:**
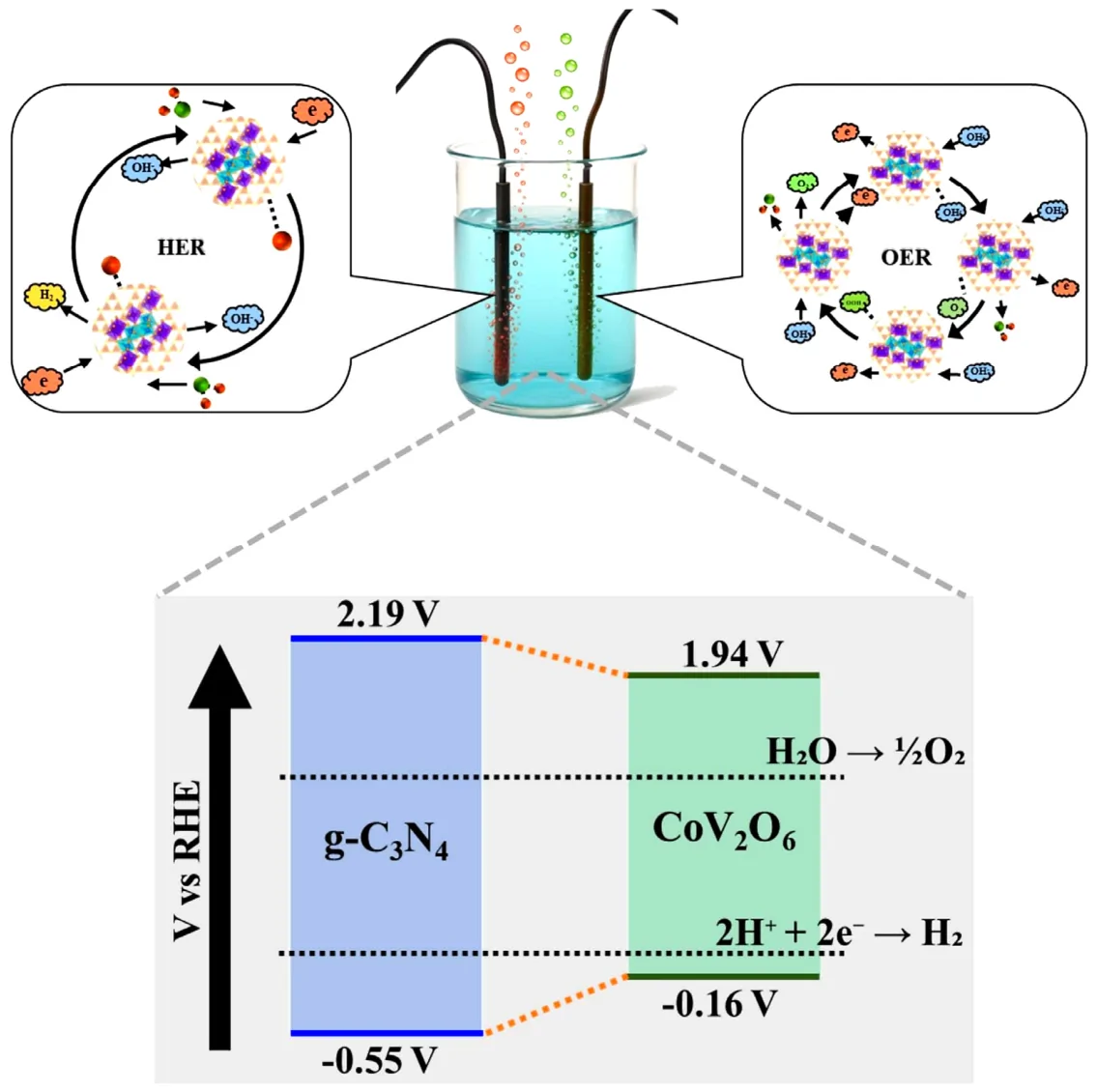
Mechanism of hydrogen and oxygen evolution using CoV_2_O_6_ as a cocatalyst in electrocatalysis, Reprinted with permission from Elsevier 2025 [66].

In contrast, CoV_2_O_6_ has primarily been investigated for magnetic materials, supercapacitors, batteries, and electrocatalytic applications. Reports concerning photocatalytic or PEC hydrogen production are essentially absent. The limited photocatalytic interest in CoV_2_O_6_ is commonly associated with strong localization of Co 3d electrons, unfavorable band-edge positions, and rapid recombination of photogenerated charge carriers. Consequently, CoV_2_O_6_ is currently regarded as a potential cocatalyst or electrochemical material rather than a visible-light photocatalyst for hydrogen evolution (Figure 11).

Overall, the current state of research indicates that hydrogen production over MV_2_O_6_-based systems remains at an early stage of development. Among the investigated compounds, MnV_2_O_6_ demonstrates the most direct photocatalytic hydrogen-evolution capability, whereas ZnV_2_O_6_ and NiV_2_O_6_ exhibit promising photoelectrochemical water-splitting performance (Table 4). Future studies should focus on heterojunction engineering, defect creation, cocatalyst loading, morphology control, and Z-scheme or S-scheme architectures to improve charge separation and maximize solar-to-hydrogen conversion efficiency.

### 5.2. Oxygen Evolution Performance

In addition to hydrogen production, oxygen evolution is a critical half-reaction in photocatalytic water splitting because it involves a complex four-electron transfer process and often governs the overall reaction kinetics. Among the MV_2_O_6_ (M = Zn, Ni, Cu, Mg, Mn, and Co) family, research on oxygen evolution has been relatively more extensive than hydrogen evolution, particularly in the form of photoelectrochemical (PEC) water oxidation. The narrow band gaps, visible-light absorption capabilities, and favorable valence-band positions of several MV_2_O_6_ compounds make them attractive candidates for oxygen evolution reactions (OER). However, the majority of studies have focused on PEC oxygen generation rather than suspended-particle photocatalytic oxygen evolution.

ZnV_2_O_6_ has attracted considerable attention as a visible-light-responsive photoanode owing to its suitable valence-band position and good photochemical stability. Several studies have demonstrated enhanced photocurrent generation through elemental doping and heterostructure formation with conductive carbon materials. In particular, Mo-doped ZnV_2_O_6_/reduced graphene oxide (rGO) photoanodes exhibited significantly improved PEC performance under simulated solar irradiation, indicating efficient hole generation and transfer for water oxidation. The enhanced oxygen-evolution activity was attributed to improved charge separation, increased carrier concentration, and accelerated interfacial charge-transfer kinetics induced by Mo incorporation and the conductive graphene framework (Figure 9). Although direct quantification of oxygen evolution was not the primary focus of these studies, the observed photocurrent enhancement strongly suggests improved OER kinetics under PEC conditions. Whether this translates to particulate photocatalysis remains to be demonstrated.

**Figure 12:**
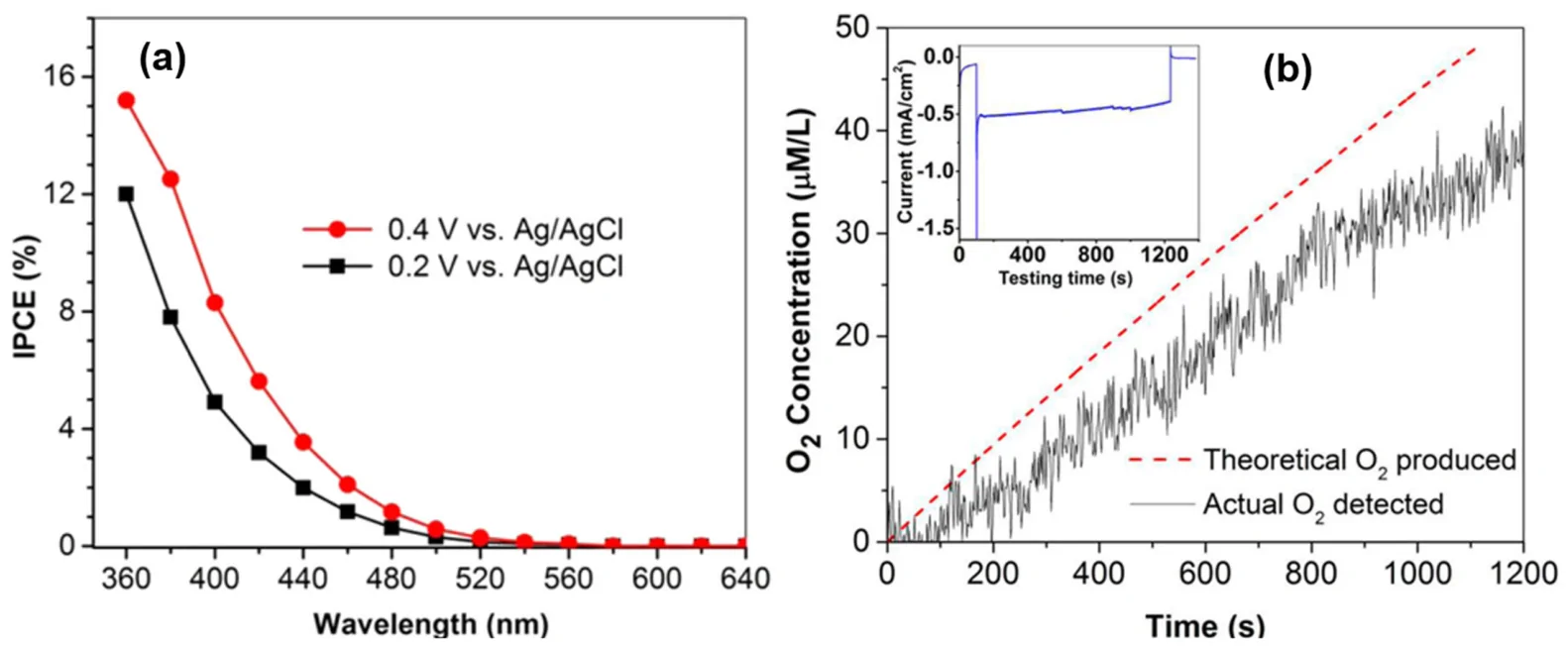
(**a**) Representative IPCE spectra of NiV_2_O_6_ films at various applied biases versus Ag/AgCl in 1 M KOH under AM1.5G illumination (∼100 mW cm^−2^) with a visible-light cutoff filter (λ ≥ 420 nm). (**b**) Oxygen evolution from a NiV_2_O_6_ film under illumination in 0.1 M borate buffer (H_2_BO_3_, pH 9) at ∼400 mW cm^−2^, with the corresponding current density shown in the inset, Reprinted with permission from ACS 2014 [67].

Among the MV_2_O_6_ family, NiV_2_O_6_ represents one of the most extensively investigated materials for oxygen evolution. Dang et al. reported that triclinic NiV_2_O_6_ thin-film photoanodes exhibit efficient visible-light-driven water oxidation with stable oxygen generation under alkaline conditions. Approximately 45% of the photocurrent originated from wavelengths exceeding 420 nm, confirming substantial visible-light utilization. Furthermore, oxygen evolution was experimentally verified with Faradaic efficiencies approaching 80%, demonstrating that photogenerated holes effectively participated in water oxidation rather than undergoing parasitic side reactions (Figure 12). The favorable performance was attributed to the narrow band gap (~2.1–2.4 eV), strong visible-light absorption, and suitable valence-band potential of NiV_2_O_6_ for driving the oxygen evolution reaction. Consequently, NiV_2_O_6_ is currently regarded as one of the most promising MV_2_O_6_ photoanodes for solar water oxidation, though particulate photocatalytic OER has not yet been demonstrated for this material.

CuV_2_O_6_ has also been investigated for photoelectrochemical water oxidation due to its strong visible-light absorption and high theoretical light-harvesting capability. The electronic structure of CuV_2_O_6_ enables efficient generation of photogenerated holes under visible-light irradiation. Nevertheless, practical oxygen-evolution performance remains limited by rapid charge-carrier recombination and relatively poor charge-transport properties. As a result, current research efforts focus on improving its PEC activity through morphology control, heterojunction construction, and cocatalyst loading. Although direct oxygen-evolution measurements remain scarce, CuV_2_O_6_ continues to be considered a promising photoanode material for visible-light-driven OER applications.

In contrast, MgV_2_O_6_ remains largely unexplored for oxygen evolution. Existing reports primarily concern crystal structure determination, optical characterization, dielectric properties, and environmental photocatalytic applications. To date, no significant studies have demonstrated photocatalytic or photoelectrochemical oxygen evolution over MgV_2_O_6_. The relatively wide band gap and limited electrical conductivity are believed to restrict its effectiveness for solar-driven water oxidation. Therefore, further investigation of its electronic structure and band-edge positions is required to assess its suitability for OER applications.

**Figure 13:**
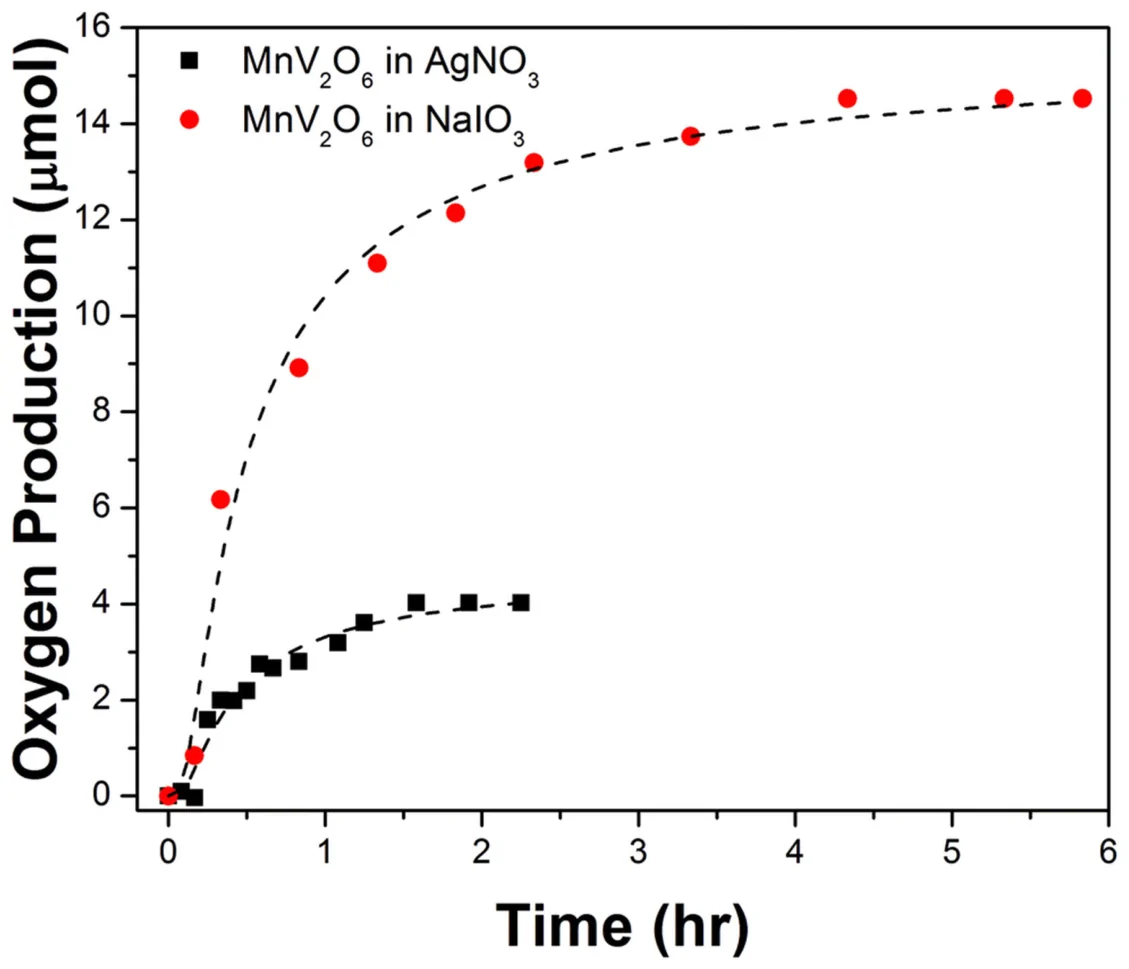
Oxygen evolution by MnV_2_O_6_ under visible-light irradiation in the presence of AgNO_3_ and NaIO_3_ as sacrificial reagents, Reprinted with permission from RSC 2017 [65].

MnV_2_O_6_ is unique within the MV_2_O_6_ family because it has demonstrated simultaneous photocatalytic evolution of both hydrogen and oxygen under visible-light irradiation. Zoellner et al. reported that p-type MnV_2_O_6_ possesses band-edge positions that thermodynamically straddle the water redox potentials, enabling overall water-splitting activity. Photocatalytic experiments confirmed the generation of oxygen alongside hydrogen, indicating that photogenerated holes were sufficiently energetic to oxidize water molecules, approximately 4 μmol of gas was produced over about 2.5 h (Figure 13). The observed activity was attributed to charge-transfer transitions between Mn 3d and V 3d orbitals, favorable band-edge alignment, and effective charge separation arising from its p-type semiconducting character. These findings establish MnV_2_O_6_ as one of the few single-phase MV_2_O_6_ compounds capable of simultaneously driving both half-reactions of water splitting.

CoV_2_O_6_ has primarily been investigated for electrochemical energy-storage devices, supercapacitors, batteries, and electrocatalytic oxygen evolution. While photocatalytic oxygen evolution studies are essentially absent, CoV_2_O_6_-derived materials have demonstrated promising electrocatalytic OER activity due to the redox-active Co^2+^/Co^3+^ centers and favorable surface reaction kinetics. Consequently, CoV_2_O_6_ may serve as a potential cocatalyst or OER-promoting component in future photocatalytic heterostructures, although direct evidence for photocatalytic oxygen evolution remains unavailable.

Overall, oxygen evolution studies on MV_2_O_6_ compounds remain dominated by photoelectrochemical investigations. NiV_2_O_6_ exhibits the most convincing PEC oxygen-evolution performance, while MnV_2_O_6_ represents the only member that has clearly demonstrated photocatalytic generation of oxygen in conjunction with hydrogen evolution (Table 5). ZnV_2_O_6_ and CuV_2_O_6_ show considerable promise as visible-light-responsive photoanodes, whereas MgV_2_O_6_ and CoV_2_O_6_ remain largely unexplored for photocatalytic oxygen production. Future research should focus on cocatalyst integration, surface oxygen-vacancy engineering, heterojunction construction, and band-structure modulation to enhance hole utilization efficiency and accelerate oxygen-evolution kinetics in MV_2_O_6_-based systems.

## 6. Requirements and Challenges for Advancing MV_2_O_6_-Based Photocatalysts

Despite the promising optical and electronic properties of MV_2_O_6_ (M = Zn, Ni, Cu, Mg, Mn, and Co) metavanadates, their application in efficient solar-driven water splitting remains at an early stage. Current studies indicate that only a few members of this family, particularly MnV_2_O_6_, have demonstrated direct photocatalytic hydrogen and oxygen evolution, while ZnV_2_O_6_, NiV_2_O_6_, and CuV_2_O_6_ have primarily been explored as photoelectrodes for water oxidation. Consequently, several fundamental and technological challenges must be addressed before MV_2_O_6_-based materials can become viable photocatalysts for practical solar fuel production.

### 6.1. Band Structure Optimization and Thermodynamic Requirements

A primary requirement for efficient photocatalytic water splitting is the appropriate alignment of the conduction-band minimum (CBM) and valence-band maximum (VBM) relative to the hydrogen and oxygen redox potentials. Although most MV_2_O_6_ compounds exhibit visible-light-responsive band gaps ranging from approximately 2.0 to 2.5 eV, their band-edge positions vary considerably depending on the transition-metal cation.

Among the reported materials, MnV_2_O_6_ possesses band-edge positions capable of thermodynamically driving both hydrogen and oxygen evolution reactions. In contrast, NiV_2_O_6_ and CuV_2_O_6_ have predominantly demonstrated water oxidation activity, suggesting that their electronic structures are more favorable for hole-driven oxidation processes than proton reduction. Similarly, ZnV_2_O_6_ exhibits promising photoelectrochemical performance but has rarely been employed as a standalone photocatalyst for hydrogen generation. These observations highlight the need for systematic band-edge engineering through cation substitution, defect modulation, and heterojunction construction to enhance reduction capabilities while maintaining strong visible-light absorption.

### 6.2. Charge Separation and Carrier-Recombination Challenges

Rapid recombination of photogenerated electron–hole pairs remains one of the major limitations affecting the photocatalytic performance of MV_2_O_6_ materials. The complex electronic interactions between transition-metal d orbitals and V–O polyhedra often result in localized charge carriers and limited carrier mobility. Consequently, a significant fraction of photogenerated charges recombine before participating in surface redox reactions.

Several studies have demonstrated that heterostructure formation, graphene incorporation, elemental doping, and defect engineering can substantially improve charge separation. For example, Mo-doped ZnV_2_O_6_/reduced graphene oxide photoanodes exhibited enhanced photocurrent densities owing to improved charge transport and reduced recombination losses. Nevertheless, the fundamental charge-transfer mechanisms within MV_2_O_6_ systems remain insufficiently understood. Future research should focus on ultrafast spectroscopic investigations, defect-state engineering, and nanoscale morphology control to maximize charge-carrier lifetimes and transport efficiencies.

### 6.3. Surface Reaction Kinetics and Cocatalyst Development

Even when efficient charge separation is achieved, the surface kinetics of hydrogen evolution reaction (HER) and oxygen evolution reaction (OER) remain challenging. In particular, oxygen evolution involves a complex four-electron transfer process that often represents the rate-limiting step in photocatalytic water splitting. Current reports indicate that NiV_2_O_6_ and ZnV_2_O_6_ function effectively as photoanodes for water oxidation; however, their overall solar-to-fuel conversion efficiencies remain limited by sluggish surface reaction kinetics.

Loading suitable cocatalysts is therefore essential for accelerating interfacial charge transfer and reducing reaction overpotentials. Noble-metal cocatalysts such as Pt, RuO_2_, and IrO_2_ remain highly effective but suffer from high cost and limited availability. Future studies should focus on earth-abundant alternatives, including transition-metal phosphides, sulfides, hydroxides, and single-atom catalysts. In addition, mechanistic investigations are needed to clarify the active sites and reaction pathways governing HER and OER on MV_2_O_6_ surfaces.

### 6.4. Suppression of Side Reactions During Photocatalytic Water Splitting

In addition to limited charge separation and sluggish surface reaction kinetics, parasitic side reactions significantly reduce the overall efficiency of photocatalytic water splitting. These competing reactions consume photogenerated charge carriers without contributing to hydrogen or oxygen production, thereby lowering the apparent quantum efficiency and solar-to-hydrogen conversion efficiency.

One of the most common side reactions is the back reaction, in which the evolved H_2_ and O_2_ recombine to form water on the photocatalyst surface, particularly in the presence of catalytically active noble metals. In addition, dissolved oxygen can compete with proton reduction by acting as an electron acceptor, while surface defects may promote undesirable redox reactions involving intermediate species instead of the targeted hydrogen evolution reaction (HER) and oxygen evolution reaction (OER). Under practical operating conditions, photocorrosion and photocatalyst self-oxidation or self-reduction may also occur, resulting in structural degradation and loss of catalytic activity during prolonged illumination.

Several strategies can effectively suppress these side reactions. Selective cocatalyst loading is one of the most effective approaches, where HER and OER cocatalysts are spatially separated to promote directional charge transfer while minimizing reverse reactions between the generated products. Heterojunction engineering, particularly through Z-scheme and S-scheme architectures, enhances charge separation and directs photogenerated electrons and holes toward their respective reaction sites, thereby reducing the probability of competing interfacial reactions. Surface passivation and protective coatings can inhibit photocorrosion while maintaining efficient charge transfer across the catalyst–electrolyte interface. Furthermore, careful defect engineering is essential because an optimal concentration of oxygen vacancies can facilitate charge transport and surface activation, whereas excessive defect densities may introduce recombination centers and promote undesirable side reactions. Finally, optimizing reaction conditions—including solution pH, cocatalyst loading, dissolved oxygen concentration, and electrolyte composition—can further suppress competing reactions and improve the selectivity of overall water splitting.

For MV_2_O_6_-based photocatalysts, systematic investigations into the relationship between surface chemistry, defect structures, and reaction selectivity remain scarce. Future studies combining operando spectroscopic techniques, isotopic labeling experiments, and theoretical calculations will be essential for identifying the dominant side reactions and developing rational strategies to suppress them. Such efforts are expected to improve both the efficiency and long-term stability of MV_2_O_6_ photocatalysts for practical solar hydrogen production.

### 6.5. Limited Exploration of Composition–Structure–Activity Relationships

Compared with other vanadate families, the photocatalytic investigation of MV_2_O_6_ compounds remains relatively limited. Current studies mainly focus on ZnV_2_O_6_, NiV_2_O_6_, and MnV_2_O_6_, whereas MgV_2_O_6_ and CoV_2_O_6_ remain largely unexplored. Furthermore, systematic correlations among composition, crystal structure, morphology, defect chemistry, and photocatalytic performance are still lacking, limiting the rational design of efficient photocatalysts.

Future research should establish composition–structure–activity relationships through systematic compositional tuning, morphology engineering, and controlled defect introduction to optimize band structures, charge separation, and surface reaction kinetics. Combining advanced characterization techniques, such as XPS, EPR, and operando spectroscopy, with density functional theory (DFT) calculations will provide valuable insights into charge-transfer mechanisms and active sites. Such integrated experimental and theoretical studies will accelerate the rational design of high-performance MV_2_O_6_ photocatalysts for efficient solar water splitting.

### 6.6. Scalability and Practical Implementation

The practical deployment of MV_2_O_6_-based photocatalysts also requires scalable and economically viable synthesis strategies. Conventional solid-state methods are attractive because of their simplicity and ease of scale-up; however, they often yield large particle sizes, low surface areas, and limited control over morphology. Hydrothermal, solvothermal, and template-assisted routes provide improved structural control but may increase processing complexity and cost.

Future large-scale production should prioritize synthesis approaches capable of simultaneously controlling crystal phase, particle size, defect concentration, and surface area while maintaining low environmental impact and cost. In addition, long-term photostability, resistance to photocorrosion, and operation under natural solar irradiation must be systematically evaluated before industrial implementation becomes feasible.

## 7. Conclusions and Future Perspectives

The growing global demand for sustainable and carbon-neutral energy technologies has intensified the search for efficient visible-light-responsive photocatalysts for solar water splitting. In this context, divalent metal metavanadates (MV_2_O_6_, where M = Zn, Ni, Cu, Mg, Mn, and Co) have emerged as a promising yet relatively underexplored family of semiconductor materials. Their narrow-to-moderate band gaps, strong visible-light absorption, tunable electronic structures, and compositional flexibility make them attractive candidates for photocatalytic and photoelectrochemical energy-conversion applications.

This review has comprehensively summarized the crystal structures, electronic properties, synthesis strategies, and photocatalytic performances of MV_2_O_6_-based systems. The available literature demonstrates that the photocatalytic behavior of these materials is strongly governed by the nature of the divalent metal cation, which influences crystal symmetry, band-edge positions, charge-carrier mobility, and redox activity. Among the investigated compounds, MnV_2_O_6_ represents the most notable example of a single-phase MV_2_O_6_ photocatalyst capable of simultaneously producing hydrogen and oxygen under visible-light irradiation. In contrast, ZnV_2_O_6_ and NiV_2_O_6_ have primarily demonstrated promising photoelectrochemical water-splitting performance, particularly as visible-light-active photoanodes for oxygen evolution. CuV_2_O_6_ has shown potential as a photoelectrode and heterojunction component due to its strong visible-light absorption, whereas MgV_2_O_6_ and CoV_2_O_6_ remain largely unexplored for photocatalytic water-splitting applications.

Despite these encouraging developments, the overall photocatalytic performance of MV_2_O_6_ materials remains significantly below the levels required for practical solar hydrogen production. Several key challenges continue to limit their efficiency, including insufficient charge separation, rapid electron–hole recombination, sluggish surface reaction kinetics, and limited understanding of composition–structure–activity relationships. Furthermore, only a small fraction of the MV_2_O_6_ family has been systematically investigated for hydrogen and oxygen evolution, leaving substantial opportunities for discovering new photocatalytic functionalities within this material class.

Future research should therefore focus on rational electronic-structure engineering to optimize band-edge positions and enhance redox driving forces. Strategies such as cation substitution, defect engineering, oxygen-vacancy creation, and controlled valence-state modulation may provide effective pathways for improving visible-light absorption and charge-carrier transport. Equally important is the development of advanced heterojunction architectures, including Type-II, Z-scheme, and S-scheme systems, which can promote efficient spatial separation of photogenerated charge carriers while preserving strong reduction and oxidation capabilities. The successful implementation of such approaches has already been demonstrated in several vanadate-based photocatalysts and is expected to play a crucial role in unlocking the full potential of MV_2_O_6_ materials.

Another promising direction involves the integration of cocatalysts to accelerate hydrogen evolution reaction (HER) and oxygen evolution reaction (OER) kinetics. While noble-metal cocatalysts such as Pt, RuO_2_, and IrO_2_ remain highly effective, future efforts should prioritize the development of earth-abundant alternatives, including transition-metal phosphides, sulfides, nitrides, hydroxides, and single-atom catalysts. Coupling these cocatalysts with optimized MV_2_O_6_ hosts may significantly reduce reaction overpotentials and improve overall solar-to-hydrogen conversion efficiencies.

From a fundamental perspective, advanced characterization techniques and theoretical investigations are required to establish clear correlations between crystal structure, electronic configuration, defect chemistry, and photocatalytic activity. In particular, in situ and operando spectroscopic methods, combined with density functional theory (DFT) calculations, can provide valuable insights into charge-transfer mechanisms, surface reaction pathways, and active catalytic sites. Such studies will be essential for guiding the rational design of next-generation MV_2_O_6_ photocatalysts.

Moreover, future investigations should move beyond individual materials and explore the entire MV_2_O_6_ compositional space through high-throughput experimental screening and computational materials discovery. The limited number of studies currently available for MgV_2_O_6_ and CoV_2_O_6_ suggests that substantial opportunities remain for uncovering previously unexplored photocatalytic properties. Similarly, mixed-cation vanadates, multicomponent heterostructures, and nanostructured architectures may offer new avenues for achieving synergistic improvements in light harvesting, charge separation, and catalytic activity.

Finally, translating laboratory-scale achievements into practical solar-fuel technologies will require greater emphasis on scalable synthesis methods, long-term photostability, photocorrosion resistance, and operation under realistic solar irradiation conditions. Future studies should therefore incorporate standardized photocatalytic testing protocols, apparent quantum efficiency measurements, and durability assessments to facilitate meaningful comparisons among different MV_2_O_6_ systems.

In summary, MV_2_O_6_ metavanadates constitute a scientifically intriguing and technologically promising class of visible-light-responsive materials for solar energy conversion. Although research in this area remains at a relatively early stage, the encouraging performance of MnV_2_O_6_, ZnV_2_O_6_, and NiV_2_O_6_ demonstrates the potential of this family for photocatalytic and photoelectrochemical water splitting. Continued advances in materials design, heterojunction engineering, cocatalyst integration, and mechanistic understanding are expected to accelerate the development of highly efficient MV_2_O_6_-based photocatalysts and contribute to the realization of sustainable solar hydrogen production technologies.

## Figures and Tables

**Table 1:** Crystallographic parameters of representative MV_2_O_6_ metavanadates.

Compound	Crystal System	Space Group	Structural Characteristics	Refs.
ZnV_2_O_6_	Monoclinic	C2/m	Edge-sharing VO_6_ octahedral chains	[43,44]
NiV_2_O_6_	Monoclinic	C2/m	Distorted VO_6_ and NiO_6_ octahedra	[45]
CoV_2_O_6_	Monoclinic	C2/m	Chain-like octahedral framework	[9,46]
MnV_2_O_6_	Monoclinic	C2/m	Distorted octahedral network	[32]
MgV_2_O_6_	Monoclinic	C2/m	Thermally stable metavanadate framework	[38]
CuV_2_O_6_	Triclinic	P1	Jahn–Teller distorted CuO_6_ octahedra	[47]

**Table 2:** Reported Electronic Properties of Representative MV_2_O_6_ Metavanadates.

Compound	Reported Band Gap (eV)	Visible-Light Response	Reported Water-Splitting Activity
ZnV_2_O_6_	~2.2–2.4	Yes	PEC water oxidation; H_2_ evolution in composite systems
CuV_2_O_6_	~1.8–2.0	Strong	PEC water oxidation; heterojunction component
NiV_2_O_6_	~2.0–2.3	Yes	Photoanode for water oxidation
CoV_2_O_6_	~2.0–2.3	Yes	Limited photocatalytic studies
MnV_2_O_6_	~1.9–2.2	Yes	Direct photocatalytic H_2_ and O_2_ evolution
MgV_2_O_6_	~2.3–2.5	Moderate	Limited reports

**Table 3:** Representative Morphologies and Synthesis Routes Reported for MV_2_O_6_ Materials.

Composition	Reported Morphology	Synthesis Route	Refs.
ZnV_2_O_6_	Nanorods, nanowires, nanobelts	Hydrothermal, microwave-assisted synthesis	[49,50,51]
CuV_2_O_6_	Nanowires, porous particles, thin films	Electrospinning, solution-based synthesis, thermal treatment	[47,52,53]
NiV_2_O_6_	Nanorods, mesoporous nanostructures, nanospheres	Hydrothermal, solution-based synthesis	[54,55]
MnV_2_O_6_	Nanobelts, nanorods, polycrystalline particles	Hydrothermal, solid-state synthesis	[32,56]
CoV_2_O_6_	Nanoflowers, nanosheets, nanoparticles	Hydrothermal, surfactant-assisted synthesis	[9,46]
MgV_2_O_6_	Polycrystalline powders, microstructured particles	Solid-state synthesis	[31,38,57]

**Table 4:** Reported Photocatalytic and Photoelectrochemical Hydrogen Production Performance of MV_2_O_6_-Based Systems.

Material	System Type	Application	Key Performance	Remarks	Ref.
ZnV_2_O_6_	Mo-doped ZnV_2_O_6_/rGO photoanode	PEC hydrogen production	2.07 mA cm^−2^ at 1.23 V vs. RHE; IPCE = 17% at 370 nm	Enhanced charge separation via Mo doping and graphene incorporation	[64]
NiV_2_O_6_	Thin-film photoanode	PEC water oxidation	~45% photocurrent from λ > 420 nm; FE ≈ 80%	Visible-light-responsive PEC photoanode	[67]
CuV_2_O_6_	Pure semiconductor	Photocatalysis/PEC	No significant H_2_ evolution reported	Mainly studied for degradation and thermochemical applications	[68,69]
MgV_2_O_6_	Pure semiconductor	Optical/photocatalytic studies	No H_2_ production reports	Largely unexplored for water splitting	[38,57]
MnV_2_O_6_	Suspended photocatalyst	Photocatalytic H_2_ and O_2_ evolution	H_2_: ~1.5 μmol h^−1^ (in 20% methanol); O_2_: ~1.6 μmol h^−1^ (with AgNO_3_/NaIO_3_).	Most convincing single-phase MV_2_O_6_ photocatalyst for H_2_ production	[65]
CoV_2_O_6_	Pure semiconductor	Electrochemical applications	No H_2_ production reports	Primarily studied for energy storage and electrocatalysis	[46,66]

**Table 5:** Reported Oxygen Evolution Performance of MV_2_O_6_-Based Systems.

Material	System Type	Oxygen Evolution Application	Key Performance	Major Findings	Ref.
ZnV_2_O_6_	Mo-doped ZnV_2_O_6_/rGO photoanode	PEC water oxidation	2.07 mA cm^−2^ at 1.23 V vs. RHE; IPCE 17% at 370 nm	Improved hole transfer and water oxidation due to Mo doping and rGO	[64]
NiV_2_O_6_	Thin-film photoanode	PEC oxygen evolution	Stable oxygen generation; Faradaic efficiency ~80%; ~45% photocurrent from λ > 420 nm; photocurrent ~0.2 mA cm^−2^ at 0.6 V vs. Ag/AgCl	Most extensively studied MV_2_O_6_ photoanode for OER	[67]
CuV_2_O_6_	Photoanode	PEC water oxidation	No quantitative photocurrent data reported	Strong visible-light absorption but significant charge recombination	[47]
MgV_2_O_6_	Pure semiconductor	OER studies	No reports	Largely unexplored	—
MnV_2_O_6_	Suspended photocatalyst	Photocatalytic oxygen evolution	O_2_: ~1.6 μmol h^−1^ (with AgNO_3_/NaIO_3_ as sacrificial reagents).	Only single-phase MV_2_O_6_ reported for direct photocatalytic O_2_ evolution	[65]
CoV_2_O_6_	Electrocatalyst	Electrochemical OER	No photocatalytic OER data reported	Photocatalytic O_2_ evolution not reported	[66]

## Data Availability

No new data were created or analyzed in this study. Data sharing is not applicable to this article.
